# A vibration control method for chatter mitigation in milling process based on sliding mode control and reinforcement learning

**DOI:** 10.1038/s41598-026-62061-w

**Published:** 2026-07-16

**Authors:** Satyam Paul

**Affiliations:** School of Science and Technology, Örebro University, Örebro, Sweden

**Keywords:** Engineering, Mathematics and computing

## Abstract

Chatter is a self-excited vibration phenomenon that limits the productivity, surface quality and tool life of milling operations. In this study, an active chatter mitigation strategy is proposed by integrating sliding mode control (SMC) with reinforcement learning (RL) and an active vibration damper (AVD). A two-degree-of-freedom milling model is first formulated to describe tool vibration in the feed and normal directions, including regenerative cutting-force effects, nonlinear force components and damper-friction dynamics. A continuous-time sliding mode controller is then developed to provide robust suppression of chatter under bounded nonlinearities and modelling uncertainties. To reduce the conservative switching action and improve adaptive compensation, an actor-critic reinforcement learning component is incorporated as a bounded auxiliary control signal. The RL agent uses vibration states, sliding variables and previous control information to learn compensation forces that reduce residual chatter while penalising excessive control effort and abrupt force variations. A Lyapunov-based boundedness theorem is established to show that the sliding surface, vibration error and closed-loop milling states remain uniformly ultimately bounded when the switching gain dominates the lumped uncertainty and bounded RL compensation. Numerical simulations are conducted using cutting and structural parameters extracted from established nonlinear milling chatter studies. The proposed SMC-RL controller is compared with an uncontrolled case and a conventional PID controller. The results show that the proposed method achieves faster vibration decay and lower residual chatter in both vibration directions. Based on the mean squared error indicator, the proposed controller achieves vibration attenuation of 86.27% in the *x*-direction and 87.09% in the *y*-direction, outperforming the PID benchmark. These findings demonstrate that combining SMC robustness with RL-based adaptive compensation provides a promising framework for active chatter suppression in high-productivity milling processes.

## Introduction

High-quality machining is a central requirement in modern manufacturing because dimensional accuracy, surface integrity, productivity and tool life are directly connected to the dynamic behaviour of the machine-tool-workpiece system. Among the dynamic phenomena that restrict machining performance, chatter remains one of the most critical limitations in milling. Chatter is a self-excited vibration that develops from the interaction between the cutting process and the structural dynamics of the machine tool. Once chatter appears, the cutting edge no longer removes material under steady conditions; instead, the tool interacts with a previously generated wavy surface and produces a new waviness that can amplify the subsequent chip thickness. This regenerative mechanism leads to poor surface finish, increased tool wear, reduced dimensional accuracy, larger acoustic emission, possible damage to spindle and fixture components, and a reduction in the allowable material removal rate. Quintana and Ciurana (2011) described chatter as a long-standing limitation to productivity and part quality in machining, while Altintas and Budak (1995) showed that stability prediction in milling depends strongly on the transfer functions at the cutter-workpiece contact zone, cutting force coefficients, radial immersion and the number of cutter teeth^1,2^. In a previous active chatter-control study, Paul and Morales-Menendez (2019) also emphasized that self-generated vibration degrades finished-product quality and causes tool wear, dimensional inaccuracy and reduced material removal rate in milling^3^.

The classical explanation of milling chatter is commonly associated with regenerative chip-thickness variation. During milling, each tooth removes material from a surface that was partly shaped by the preceding tooth. If the dynamic phase between the current and previous tool positions becomes unfavourable, the instantaneous chip thickness varies periodically and the cutting force can inject energy into the structural modes of the machine-tool system. Stability lobe diagrams have therefore been widely used to select spindle speed and axial depth of cut combinations that avoid unstable regenerative conditions. Altintas and Budak (1995) developed an analytical prediction method for milling stability lobes, and this approach remains a key foundation for process planning^1^. However, stability-lobe-based selection is mainly a process-parameter avoidance method: it prevents chatter by restricting the cutting parameters to stable regions rather than actively suppressing vibration when the process moves into unstable or near-critical conditions. This is especially important because industrial productivity normally demands large depths of cut, high spindle speeds and flexible tooling or workpiece setups that may operate close to the stability boundary.

Existing chatter mitigation methods may be grouped into three broad categories. The first category is based on stability prediction and selection of chatter-free machining parameters, including stability lobe diagrams and related frequency-domain or time-domain methods. The second category modifies the regenerative mechanism by changing spindle speed or cutting parameters, for example through spindle speed variation. The third category modifies the structural dynamics of the machining system by passive, semi-active or active vibration-control methods. Paul and Morales-Menendez (2019) used this classification and noted that passive devices such as tuned mass dampers and dynamic vibration absorbers can be attractive because of their low cost and installation simplicity, but they normally require tuning around specific frequencies and may lose effectiveness when machining conditions change^3^. Moradi et al. (2012) demonstrated that tunable vibration absorbers can improve nonlinear milling stability, but such approaches still depend on the ability to adjust or tune absorber characteristics with respect to the evolving process dynamics^4^. These issues motivate active control strategies where sensors, actuators and control algorithms are used to modify the system response during machining.

Active chatter suppression has been studied through different actuator and controller combinations. Dohner et al. (2004) investigated the mitigation of chatter instabilities in milling by active structural control, providing an important early demonstration that active actuation can enlarge stable machining regions^5^. Chen et al. (2014) proposed adaptive active chatter control in milling processes using a Fourier-series-based representation to compensate the regenerative effect^6^. Paul and Morales-Menendez (2019) later proposed an active vibration damper (AVD)-based milling chatter mitigation method in which the AVD is mounted on the spindle top and the nonlinear milling model is controlled using discrete-time sliding mode control combined with type-2 fuzzy logic^3^. The use of an AVD is attractive for the present study because it provides a physical mechanism for imposing control forces in the tool-vibration directions without directly altering the cutting parameters. In this paper, the same general AVD concept is retained, but the controller is redesigned using continuous-time sliding mode control (SMC) and reinforcement learning (RL) to improve adaptivity, reduce control chattering and preserve robustness against nonlinear cutting-force and damper-friction effects.

The control of milling chatter is challenging because the cutting process is nonlinear, time-delayed and uncertain. Cutting forces may include size effects, nonlinear force coefficients, nonlinear friction and regenerative time delay. Melkote and Endres (1998) showed that size effects are important in slot milling force modelling, particularly when chip thickness becomes comparable with the cutting-edge radius^7^. Moradi et al. (2012) represented nonlinear milling forces using polynomial terms that include linear, quadratic, cubic and coupled chip-thickness components, and Paul and Morales-Menendez (2019) used this type of nonlinear force structure in their milling chatter-control model^3,4^. For an active controller, these nonlinearities are not merely modelling details; they appear as uncertainty and disturbance terms in the closed-loop system. A controller that performs well only for a fixed linear model may therefore become ineffective when tool wear, cutting conditions, spindle speed, radial immersion or damper friction changes. This motivates the use of robust and adaptive intelligent control methods.

Sliding mode control is a strong candidate for chatter suppression because it is designed for uncertain nonlinear systems and can enforce desired error dynamics through a switching action. In vibration-control applications, SMC is attractive because the sliding surface can be chosen from displacement and velocity errors, which directly represent vibration states. SMC is suitable for milling-tool vibration attenuation because it is a robust variable-structure control method with low sensitivity to model uncertainty, parameter variations and external disturbances^8,9^. Thenozhi and Yu (2016) also explained that SMC is widely used in structural vibration control because it is relatively simple and robust, but its direct implementation may lead to high-frequency chattering because the control signal changes sign rapidly near the sliding surface and because the switching gain must be large enough to dominate uncertainty bounds^10^. The chattering problem is particularly undesirable in active vibration systems because high-frequency switching can excite unmodelled dynamics, increase actuator energy consumption and reduce actuator life. In milling, where the actuator is connected to the spindle or tool structure, excessive control chattering may also introduce unwanted high-frequency dynamics into the machining process.

Several methods have been used to reduce SMC chattering. Boundary-layer methods replace the discontinuous sign function with a saturation or smooth approximation. Fuzzy sliding surfaces and adaptive switching-gain methods can also reduce high-frequency switching. Thenozhi and Yu (2016) proposed a fuzzy sliding surface and a dead-zone adaptive law for tuning the SMC switching gain, and their Lyapunov analysis showed bounded trajectories and convergence to a real sliding surface, i.e., a small bounded neighbourhood of the ideal sliding manifold^10^. Qiu and Chen (2025) similarly noted that conventional SMC suffers from chattering related to switching gain and proposed a sliding mode reinforcement learning control method where the sliding surface is used as the RL state input and an asynchronous advantage actor-critic algorithm learns a robust vibration-control policy^11^. These ideas suggest that the robustness of SMC can be preserved while the discontinuous or high-gain part of the controller is made adaptive or learned.

Reinforcement learning provides a promising route for improving active vibration-control performance under uncertain and nonlinear conditions. In RL, an agent interacts with an environment, observes states, applies actions and receives rewards that encode the control objective. Mnih et al. (2015) demonstrated that deep reinforcement learning can learn control policies from high-dimensional inputs, while Mnih et al. (2016) proposed asynchronous actor-critic learning, in which multiple parallel actor-learners improve training stability and efficiency^12,13^. For mechanical vibration control, Long et al. (2021) proposed a combined controller for a hybrid-structured flexible manipulator in which a nominal model-based sliding mode controller produced the main driving torque and an actor-critic RL controller generated a small compensation torque to suppress elastic vibration^14^. Qiu and Chen (2025) extended this direction by combining a sliding surface with an A3C learning structure for vibration control of a translational coupled double flexible beam system; their study used an identified model for pre-learning and compared the method with PD control and conventional SMC^11^. These studies provide an implementation foundation for combining SMC and RL in milling chatter suppression.

Recent robust learning-control studies further show that actor-critic RL can be combined with robust nonlinear control to address tracking, optimality and disturbance compensation in uncertain dynamic systems. Dao and Phung^15^ proposed a RISE-based actor-critic RL controller for a perturbed three-wheeled Mecanum mobile robot, where the control objective was to unify trajectory tracking, optimality and observer effectiveness under slipping disturbances. Dao et al.^16^ developed an adaptive optimal coordination controller for bilateral teleoperators with variable time delays, where actor-critic RL was combined with a RISE term to improve coordination tracking and learning convergence. These recent studies are important because they demonstrate the growing relevance of robust actor-critic learning for uncertain tracking systems. However, their control objectives are mainly trajectory tracking or coordination tracking in robotic systems, while the present study addresses regenerative chatter suppression in milling, where the control objective is to drive the tool vibration toward the chatter-free equilibrium. Moreover, the proposed method uses SMC as the main robustness-preserving component and introduces RL only as a bounded auxiliary compensation term. This differs from tracking-oriented RISE-RL frameworks because the present controller is designed for nonlinear cutting-force variation, regenerative delay, damper friction and active vibration damper force constraints in milling. Therefore, the superiority of the proposed SMC-RL method lies in adapting robust learning-based control to the specific chatter-suppression problem while preserving a clear Lyapunov-based boundedness structure and validating the method against uncontrolled, PID and standard SMC baselines.

A conventional proportional-integral-derivative (PID) controller is an important baseline because of its simplicity, industrial familiarity and low implementation cost. Nevertheless, chatter suppression is not a simple set-point regulation problem. The dynamics include regenerative delay, spindle-speed-dependent excitation, cutting-force nonlinearities and changing structural compliance. A PID controller can reduce vibration when the operating condition is close to the tuning point, but it does not explicitly account for uncertainty bounds, delayed cutting-force feedback or nonlinear damper friction. Paul and Morales-Menendez (2019) used discrete-time PID as a benchmark for active milling chatter control and showed that more advanced robust/intelligent controllers can provide stronger attenuation when nonlinearities are included in the model^3^. Therefore, the present paper retains PID as the main comparison controller, while the proposed SMC-RL method is designed to address nonlinear and uncertain vibration behaviour more systematically.

Another motivation for the present work is the need to reduce dependence on pre-defined fuzzy-rule structures. Fuzzy chatter suppression has been investigated in end milling, for example by Liang et al. (2004), who used vibration energy and frequency-spectrum features as indicators for a fuzzy controller that adjusted machining parameters^17^. Fuzzy logic is useful because it can encode expert knowledge and nonlinear mappings, but its performance depends on the design of membership functions, rules and scaling factors. In contrast, reinforcement learning can improve its policy through interaction with a simulated or identified environment. This does not remove the need for careful training and safety constraints, but it provides a route for adaptive compensation when the exact relation between vibration state, cutting force and control action is difficult to express analytically. The proposed approach therefore keeps the rigorous structure of SMC and uses RL only as a bounded auxiliary mechanism rather than as an unconstrained stand-alone controller.

The present paper proposes an active chatter mitigation method for milling based on SMC blended with RL. The proposed framework differs from the previous works in three main ways. First, a continuous-time SMC formulation is adopted so that the design can be connected directly to the physical continuous dynamics of milling vibration and to Lyapunov-based boundedness analysis. Second, instead of using fuzzy rules to compensate nonlinearities, the RL module is used as an adaptive compensation mechanism that learns from vibration-related states and the sliding surface. Third, the AVD is retained as the active actuation mechanism, but the control signal is generated by the combined action of a robust SMC term and an RL compensation term. This structure is inspired by Long et al. (2021), where SMC provides the main robust control action and RL provides auxiliary compensation, and by Qiu and Chen (2025), where the sliding surface is used as an informative RL state for vibration suppression^11,14^.

The main hypothesis is that an SMC-RL controller can reduce milling chatter more effectively than a conventional PID controller while also reducing the control chattering associated with standard SMC. The SMC part provides robustness against bounded nonlinear cutting-force uncertainty, regenerative time-delay effects and damper-friction disturbances. The RL part provides adaptive compensation by learning a mapping from vibration/sliding states to a bounded auxiliary control force or gain correction. To retain mathematical tractability, the RL output is assumed to be bounded by actuator limits or a saturation layer, and this assumption is incorporated into the stability analysis. The closed-loop proof will be developed using a Lyapunov candidate based on the sliding surface, showing that the system trajectories remain bounded and converge to a small neighbourhood of the desired chatter-free state under appropriate gain conditions. This approach follows the logic used by Thenozhi and Yu (2016), who formulated boundedness of SMC with adaptive/fuzzy modification using Lyapunov theory, but extends the compensation mechanism toward RL-based active chatter suppression^10^.

The contributions of the paper are as follows. First, a nonlinear two-degree-of-freedom milling chatter model with regenerative cutting forces, damper friction and AVD actuation is formulated in a control-ready state-space form. Second, a combined SMC-RL control architecture is proposed for active milling chatter suppression, where the SMC term provides robust convergence and the RL term supplies bounded compensation for unmodelled nonlinearities and chattering reduction. Third, a Lyapunov-based theorem is proposed to show boundedness of the closed-loop system under bounded cutting-force uncertainty, bounded damper friction and bounded RL output. Fourth, the proposed controller is designed to be compared with a conventional PID controller, and optionally with standard SMC, using vibration attenuation, mean-squared error, peak response, settling behaviour and control effort as performance indicators. The mathematical modelling required for this controller is developed in the next section.

## Mathematical modelling of the milling process with active vibration control

### Control objective, challenges and comparison framework

Before formulating the milling dynamics, the precise control objective of this study is defined. The aim is to design an active vibration damper (AVD) control force $u_c(t)$ that suppresses regenerative chatter vibration of the milling tool in both principal vibration directions. Since the desired chatter-free operating condition corresponds to zero relative vibration around the nominal cutting equilibrium, the desired displacement and velocity are selected as

1 $$\begin{aligned} x_d(t)=0,\qquad y_d(t)=0,\qquad \dot{x}_d(t)=0,\qquad \dot{y}_d(t)=0. \end{aligned}$$

It should be noted that the tracking problem considered in this work is different from general time-varying trajectory tracking in mobile robots or teleoperator systems. In the present milling application, the desired trajectory is the chatter-free equilibrium, and the control objective is therefore formulated as zero-reference vibration tracking or regulation. The tool displacement and velocity responses are required to converge toward a small bounded neighbourhood of the origin while the control force remains admissible. This formulation is suitable for milling chatter mitigation because the aim is not to follow a prescribed motion trajectory but to suppress regenerative vibration around the nominal cutting equilibrium. Thus, the control objective is to drive the tool vibration displacement vector $x_c(t)=[x(t);y(t)]^T$ and the velocity vector $\dot{x}_c(t)=[\dot{x}(t);\dot{y}(t)]^T$ toward a small bounded neighbourhood of the origin while maintaining a feasible and smooth AVD control effort.

The control problem can be expressed through the following performance objective:

2 $$\begin{aligned} \min _{u_c(t)} J = \int _{t_0}^{t_f} \left[ x_c^{T}(t)Q_xx_c(t) + \dot{x}_c^{T}(t)Q_v\dot{x}_c(t) + u_c^{T}(t)R_uu_c(t) + \Delta u_c^{T}(t)R_{\Delta }\Delta u_c(t) \right] dt. \end{aligned}$$

where $Q_x>0$ and $Q_v>0$ penalise displacement and velocity vibration responses, $R_u>0$ penalises actuator effort, and $R_{\Delta }>0$ penalises rapid changes in the control signal. The term $\Delta u_c(t)=u_c(t)-u_c(t-\Delta t)$ is included because excessive high-frequency variation in the control force may excite unmodelled actuator or spindle dynamics. Therefore, the objective is not only to reduce vibration amplitude but also to obtain a practically implementable control signal.

The main challenge is that milling chatter is nonlinear, time-delayed and uncertain. The regenerative mechanism depends on the difference between the current and delayed tool positions, which introduces the terms $x_c(t)-x_c(t-\tau )$ into the cutting-force model. In addition, nonlinear cutting-force coefficients, damper friction, actuator limitations and parameter variations enter the closed-loop dynamics as disturbances or modelling uncertainty. These issues make chatter suppression more difficult than a conventional set-point regulation problem. Stability-lobe-based approaches can avoid chatter by selecting stable cutting conditions, but they do not actively suppress vibration once the process operates near or beyond the stability boundary^1,2^. Passive or tunable absorbers can improve the dynamic response, but their effectiveness depends on proper tuning and may decrease when machining conditions change^4^. These limitations motivate the use of active and robust control.

The proposed controller is designed to address these challenges by combining sliding mode control and reinforcement learning. The SMC component provides robustness against bounded nonlinearities and matched disturbances, while the RL component learns a bounded auxiliary compensation signal from the vibration states and sliding variables. This structure is consistent with the idea of using a robust model-based controller as the main stabilising component and a learning-based controller as an adaptive compensation mechanism^11,14^. In the present work, the total control input is therefore decomposed as

3 $$\begin{aligned} u_c(t)=u_{\textrm{SMC}}(t)+u_{\textrm{RL}}(t), \end{aligned}$$

where $u_{\textrm{SMC}}(t)$ is the robust sliding-mode control force and $u_{\textrm{RL}}(t)$ is the bounded reinforcement-learning compensation force.

To evaluate whether the proposed controller meets the stated objective, four comparative cases are considered in the numerical validation. The first case is the uncontrolled milling response, which represents the reference chatter condition. The second case is a conventional PID controller, which provides an industrially familiar linear feedback benchmark. The third case is a standard SMC controller without RL compensation, which isolates the performance of the robust sliding-mode baseline. The fourth case is the proposed SMC–RL controller, which evaluates the added benefit of the bounded learning-based compensation. The comparison is performed using time-domain vibration responses, mean squared error, percentage attenuation, control-force behaviour and reward convergence. This comparison framework allows the effectiveness of the proposed method to be evaluated not only against PID control but also against standard SMC, thereby clarifying the contribution of the RL component.

### Two-degree-of-freedom milling-tool dynamics

The following assumptions are adopted to keep the model suitable for controller synthesis while retaining the key physical mechanisms of chatter. First, the dominant compliance of the spindle-tool assembly is represented by two orthogonal translational modes at the cutter centre. Second, the workpiece is assumed to be rigid compared with the tool/spindle compliance over the considered operating region. Third, the cutting-force coefficients are treated as constant during a simulation run, while their uncertainty is included in the lumped disturbance term used for robust control. Fourth, the tooth-passing delay is determined by the spindle speed and number of teeth, and runout effects are neglected in the nominal model. Fifth, the AVD dynamics are represented at the force-input level; actuator saturation and bandwidth limits will be included later in the controller implementation and simulation settings. These assumptions are consistent with common milling chatter models, where structural dynamics, regenerative delay and cutting coefficients form the essential basis for stability and control analysis^1^.

The milling process is represented by a two-degree-of-freedom tool vibration model in the transverse directions of the cutter. This modelling choice is commonly used in milling chatter studies because the dominant dynamic compliance at the cutter-workpiece contact point can often be projected into two orthogonal directions. The workpiece is assumed to be comparatively rigid with respect to the flexible tool/spindle assembly, and the cutter has $N_t$ uniformly spaced teeth. Following the active chatter-control formulation of Paul and Morales-Menendez (2019), the displacement vector of the tool centre is defined as

4 $$\begin{aligned} x_c(t)=\begin{bmatrix}x(t)&y(t)\end{bmatrix}^{T}, \end{aligned}$$

where *x*(*t*) and *y*(*t*) denote tool displacements in the two principal directions of vibration. The uncontrolled structural dynamics of the tool can be expressed as

5 $$\begin{aligned} M_m \ddot{x}_c(t)+C_m \dot{x}_c(t)+K_m x_c(t)=F_m(t), \end{aligned}$$

where $M_m$, $C_m$ and $K_m$ are the equivalent mass, damping and stiffness matrices of the milling structure, and $F_m(t)$ is the cutting-force vector acting on the tool. This second-order representation follows the two-degree-of-freedom milling model and is consistent with classical machine-tool vibration modelling in milling stability studies^1,3^. For the present work, the equivalent mass, damping and stiffness matrices are considered in diagonal form as

6 $$\begin{aligned} \begin{aligned} M_m&= \begin{bmatrix} m_x & 0 \\ 0 & m_y \end{bmatrix}, \\ C_m&= \begin{bmatrix} c_x & 0 \\ 0 & c_y \end{bmatrix}, \\ K_m&= \begin{bmatrix} k_x & 0 \\ 0 & k_y \end{bmatrix}. \end{aligned} \end{aligned}$$

where $m_x$ and $m_y$ are the equivalent modal masses, $c_x$ and $c_y$ are the equivalent damping coefficients, and $k_x$ and $k_y$ are the equivalent stiffness coefficients in the *x*- and *y*-directions, respectively. This two-degree-of-freedom diagonal representation is commonly used for milling chatter modelling when the dominant tool dynamics are resolved along two orthogonal vibration directions^1,3^. The cutting-force vector is defined as

7 $$\begin{aligned} F_m(t)=\begin{bmatrix}F_{fx}(t)&F_{fy}(t)\end{bmatrix}^{T}, \end{aligned}$$

where $F_{fx}(t)$ and $F_{fy}(t)$ are the resolved cutting-force components.

Expanding Eq. (5) along the two vibration axes gives

8 $$\begin{aligned} m_x \ddot{x}(t)+c_x\dot{x}(t)+k_xx(t)=F_{fx}(t), \end{aligned}$$

9 $$\begin{aligned} m_y \ddot{y}(t)+c_y\dot{y}(t)+k_yy(t)=F_{fy}(t). \end{aligned}$$

Equations (8) and (9) provide the basic dynamic relation between cutting excitation and tool vibration. In a stable cutting condition, the vibration amplitude remains bounded and the tool motion decays according to the structural damping. In chatter, the regenerative cutting force injects energy into the tool dynamics and can overcome the natural damping of the system. Therefore, the force model must represent the effect of delayed tool motion and nonlinear chip-thickness variation.

### Regenerative displacement and time delay

In milling, the current chip thickness depends on the difference between the present tool position and the surface left by a previous tooth. If the spindle angular speed is $\Omega$ in rad/s and the cutter has $N_t$ teeth, the tooth-passing delay is

10 $$\begin{aligned} \tau =\frac{2\pi }{N_t\Omega }. \end{aligned}$$

This delay relation is widely used in regenerative milling models^4^ and is also used in the nonlinear milling model^3^. The regenerative displacement terms are defined as

11 $$\begin{aligned} \Delta x(t)=x(t)-x(t-\tau ), \quad \Delta y(t)=y(t)-y(t-\tau ), \end{aligned}$$

where $x(t-\tau )$ and $y(t-\tau )$ are the delayed tool positions associated with the previous tooth passage. These delayed terms are responsible for the principal regenerative feedback mechanism of chatter. If the phase relationship between $x_c(t)$ and $x_c(t-\tau )$ is destabilising, the cutting force can amplify vibration from one tooth passage to the next.

The nominal milling model assumes that the cutter engagement is constant during the simulation interval and that the start and exit immersion angles are known. For half-immersion up-milling, start and exit immersion angles of 0 and $\pi /2$ are used, respectively^3^. In the present formulation, the detailed immersion-dependent coefficients are grouped into polynomial force coefficients so that the model can be used directly for SMC-RL design. This avoids unnecessary repetition of coefficient-expansion algebra while retaining the nonlinear cutting-force structure required for controller development.

### Nonlinear cutting-force model

The nonlinear cutting-force model is written as a third-order polynomial in the regenerative displacement variables. This structure is motivated by the nonlinear milling-force formulation of Moradi et al. (2012), where linear, quadratic, cubic and coupled terms are used to describe nonlinear cutting effects^4^. The resolved force in the *x* direction is expressed as

12 $$\begin{aligned} F_{fx}(t) = \frac{N_t}{2\pi }\big [&a_0+a_1\Delta x+a_2\Delta y+a_3\Delta x^2+a_4\Delta y^2+a_5\Delta x\Delta y \nonumber \\&+a_6\Delta x^3+a_7\Delta y^3+a_8\Delta x^2\Delta y+a_9\Delta x\Delta y^2 \big ], \end{aligned}$$

where $a_0,\ldots ,a_9$ are cutting-force coefficients in the *x* direction. Similarly, the resolved force in the *y* direction is written as

13 $$\begin{aligned} F_{fy}(t) = -\frac{N_t}{2\pi }\big [&b_0+b_1\Delta x+b_2\Delta y+b_3\Delta x^2+b_4\Delta y^2+b_5\Delta x\Delta y \nonumber \\&+b_6\Delta x^3+b_7\Delta y^3+b_8\Delta x^2\Delta y+b_9\Delta x\Delta y^2 \big ], \end{aligned}$$

where $b_0,\ldots ,b_9$ are cutting-force coefficients in the *y* direction. The opposite sign convention in Eq. (13) reflects the direction of the resolved cutting-force component and follows the sign structure commonly adopted in half-immersion milling-force models^18,19^. The compact coefficient notation in Eqs. (12), (13) represents the same physical effects as the expanded coefficient expressions used in nonlinear milling studies: $a_1,a_2,b_1,b_2$ capture linear regenerative stiffness-like effects; $a_3,a_4,a_5,b_3,b_4,b_5$ capture quadratic nonlinearities; and $a_6,a_7,a_8,a_9,b_6,b_7,b_8,b_9$ capture cubic and coupled nonlinearities. Melkote and Endres (1998) also showed that cutting-force models may require nonlinear size-effect terms when chip thickness becomes small, supporting the need for nonlinear force representation in milling models^7^.

For controller synthesis, Eqs. (12) and (13) can be separated into a nominal linear regenerative component and a nonlinear residual component:

14 $$\begin{aligned} F_m(t)=K_r\Delta x_c(t)+F_{nl}(\Delta x_c,t), \end{aligned}$$

where $\Delta x_c(t)=x_c(t)-x_c(t-\tau )$, $K_r\in \mathbb {R}^{2\times 2}$ is the nominal regenerative cutting matrix, and $F_{nl}$ contains the higher-order polynomial and uncertain residual terms. This decomposition is useful because the linear part explains the dominant regenerative stiffness effect, whereas $F_{nl}$ can be treated as a bounded nonlinear uncertainty to be handled by the robust SMC term and the adaptive RL compensation. The boundedness assumption is physically reasonable over a finite operating region because tool displacement, spindle speed, immersion and actuator limits are bounded in practical milling experiments.

The decomposition in Eq. (14) is also useful for interpreting the role of the proposed learning component. The SMC term is not expected to identify every cutting-force coefficient online. Instead, it is designed to provide a robust stabilising action when the total unmodelled force is bounded. The RL term can then learn a corrective action that reduces the residual vibration observed after the nominal SMC action. This separation is similar in spirit to the SMC-RL architecture of Long et al. (2021), where model-based SMC provides the main torque and actor-critic RL supplies a smaller compensation torque^14^. For milling, this is important because the cutting-force function can change with tool wear, radial immersion, spindle speed and material behaviour, whereas the vibration response and sliding surface provide real-time information about the resulting dynamic error.

### Active vibration damper model

To actively control chatter, an AVD is mounted on the spindle structure so that it can impose a control force on the tool vibration dynamics. The AVD concept follows the active-control configuration used by Paul and Morales-Menendez (2019), where the damper is placed on the spindle top and its action is resolved into the two vibration directions^3^. Active structural control has also been used in milling chatter studies by Dohner et al. (2004), who demonstrated that active control can mitigate chatter instabilities in milling^5^. The active vibration damper (AVD) is mounted on the top surface of the spindle in order to suppress chatter vibrations induced by the external cutting forces. The basic operating principle of the AVD is based on a linear servo-actuation mechanism, in which the rotary motion of the motor is transformed into the required linear control motion. Through this arrangement, the damper is able to generate control forces that counteract the undesirable tool vibration response in the principal vibration directions.

**Fig. 1:**
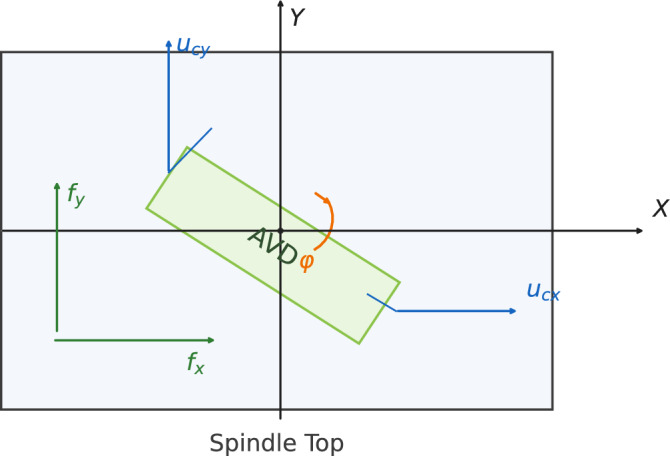
Conceptual placement of the active vibration damper (AVD) on the spindle top. The external cutting-force components $f_x$ and $f_y$ excite the milling system, while the AVD generates the control-force components $u_{cx}$ and $u_{cy}$. The damper is mounted with an inclination angle $\varphi$ to provide effective vibration suppression in both principal directions.

As illustrated in Fig. 1, the AVD is positioned close to the centre of mass of the spindle top and is oriented with an inclination angle $\varphi$. This inclined placement is advantageous because it enables the damper to influence the dynamic behaviour in both the *x*- and *y*-directions while also alleviating space limitations around the spindle assembly. Consequently, a single properly oriented AVD can provide the required control action in two directions, thereby reducing the need for multiple dampers. This leads to a more compact and cost-effective implementation of the active chatter-control system.

In the present study, the external cutting-force components $f_x$ and $f_y$ excite the milling tool-spindle system, whereas the AVD produces the control-force components $u_{cx}$ and $u_{cy}$ to mitigate the resulting vibration. The orientation angle $\varphi$ determines how the actuation force is distributed between the two principal axes. Therefore, the AVD model serves as an essential element in the proposed control framework, since it establishes the physical mechanism through which the SMC-RL controller acts on the milling system to attenuate chatter.

With the AVD included, Eq. (5) becomes

15 $$\begin{aligned} M_m \ddot{x}_c(t)+C_m\dot{x}_c(t)+K_mx_c(t)=F_m(t)+u_c(t)-d_c(t), \end{aligned}$$

where $$u_c(t)=\begin{bmatrix}u_x(t)&u_y(t)\end{bmatrix}^{T}$$ is the control-force vector generated by the AVD and $$d_c(t)=\begin{bmatrix}d_x(t)&d_y(t)\end{bmatrix}^{T}$$ represents damper friction and internal damping effects. The controlled scalar equations are therefore

16 $$\begin{aligned} m_x \ddot{x}(t)+c_x\dot{x}(t)+k_xx(t)=F_{fx}(t)+u_x(t)-d_x(t), \end{aligned}$$

17 $$\begin{aligned} m_y \ddot{y}(t)+c_y\dot{y}(t)+k_yy(t)=F_{fy}(t)+u_y(t)-d_y(t). \end{aligned}$$

The AVD is modelled as a servo-actuated moving mass. Let $m_d$ denote the moving mass of the damper and let $q_d(t)$ denote its relative displacement along the damper axis. If the damper is installed at an angle $\varphi$ with respect to the *x* axis, the damper-axis acceleration can be projected into the milling-tool vibration axes. A simplified resolved control-force relation is

18 $$\begin{aligned} u_c(t)=R_{\varphi } f_d(t), \quad R_{\varphi }=\begin{bmatrix}\cos \varphi \\ \sin \varphi \end{bmatrix}, \end{aligned}$$

where $f_d(t)$ is the commanded damper force along the damper axis. If independent equivalent force commands are used in the two projected directions, the control vector can be represented directly as

19 $$\begin{aligned} u_c(t)=\begin{bmatrix}m_d\ddot{q}_{d,x}(t)&m_d\ddot{q}_{d,y}(t)\end{bmatrix}^{T}, \end{aligned}$$

where $\ddot{q}_{d,x}$ and $\ddot{q}_{d,y}$ are the equivalent damper accelerations resolved along the *x* and *y* axes. Equation (19) is consistent with the AVD modelling approach used by Paul and Morales-Menendez (2019), in which the control action is resolved into the two milling-vibration directions^3^. The inclined-damper interpretation is useful from a design viewpoint because a single actuator can contribute to vibration suppression in both axes, reducing mechanical complexity compared with the use of two separate dampers.

### Damper friction and bounded disturbance representation

The damper introduces friction and internal damping that must be included in the control model. Following the smooth Coulomb-friction representation used in the AVD-based milling model of^20^, the damper disturbance is modelled component-wise as

20 $$\begin{aligned} d_x(t)=\Lambda \dot{q}_{d,x}(t)+\Gamma m_d g\tanh \left( \Upsilon \dot{q}_{d,x}(t)\right) , \end{aligned}$$

21 $$\begin{aligned} d_y(t)=\Lambda \dot{q}_{d,y}(t)+\Gamma m_d g\tanh \left( \Upsilon \dot{q}_{d,y}(t)\right) , \end{aligned}$$

where $\Lambda$ is a viscous damping coefficient, $\Gamma$ is a Coulomb-friction coefficient, *g* is gravitational acceleration and $\Upsilon$ determines the slope of the smooth hyperbolic tangent approximation. The use of $\tanh (\cdot )$ avoids the ideal discontinuity of a pure sign function and is compatible with numerical simulation and control design. This friction form is important because friction enters the same channel as the cutting-force disturbance and can therefore affect the AVD control effort.

Combining the nonlinear cutting-force model and damper-friction model, the controlled milling dynamics can be written as

22 $$\begin{aligned} M_m \ddot{x}_c(t)+C_m\dot{x}_c(t)+K_mx_c(t)=K_r\Delta x_c(t)+F_{nl}(\Delta x_c,t)+u_c(t)-d_c(t). \end{aligned}$$

For robust control design, the nonlinear and uncertain terms are grouped into a single disturbance vector

23 $$\begin{aligned} \Psi (t)=K_r\Delta x_c(t)+F_{nl}(\Delta x_c,t)-d_c(t)+\Delta _m(t), \end{aligned}$$

where $\Delta _m(t)$ represents modelling uncertainty, parameter variation and unmodelled dynamics. Then Eq. (22) becomes

24 $$\begin{aligned} M_m \ddot{x}_c(t)+C_m\dot{x}_c(t)+K_mx_c(t)=u_c(t)+\Psi (t). \end{aligned}$$

The following boundedness assumption is introduced for controller development:

25 $$\begin{aligned} \Vert \Psi (t)\Vert \le \bar{\Psi }, \quad \forall t\ge 0, \end{aligned}$$

where $\bar{\Psi }$ is a finite positive constant. Similar bounded-uncertainty assumptions are standard in SMC-based vibration control because the switching or robust term must dominate the uncertainty bound to guarantee convergence or ultimate boundedness. Thenozhi and Yu (2016) used bounded uncertainty assumptions in the Lyapunov analysis of sliding mode vibration control, and Qiu and Chen (2025) similarly discussed SMC robustness with respect to model uncertainties and external disturbances^11,10^.

### State-space representation for SMC-RL design

To design the proposed SMC-RL controller, Eq. (24) is converted into first-order state-space form. Define the state vector

26 $$\begin{aligned} z(t)=\begin{bmatrix}x_c^T(t)&\dot{x}_c^T(t)\end{bmatrix}^{T} =\begin{bmatrix}x(t)&y(t)&\dot{x}(t)&\dot{y}(t)\end{bmatrix}^{T}. \end{aligned}$$

The state-space model is

27 $$\begin{aligned} \dot{z}(t)=Az(t)+Bu_c(t)+B\Psi (t), \end{aligned}$$

where

28 $$\begin{aligned} A=\begin{bmatrix}0_{2\times 2}& I_{2\times 2}\\ -M_m^{-1}K_m& -M_m^{-1}C_m\end{bmatrix}, \quad B=\begin{bmatrix}0_{2\times 2}\\ M_m^{-1}\end{bmatrix}. \end{aligned}$$

The delayed regenerative contribution is contained in $\Psi (t)$ through $\Delta x_c(t)=x_c(t)-x_c(t-\tau )$. If desired, Eq. (27) may be written explicitly as a delay differential equation,

29 $$\begin{aligned} \dot{z}(t)=Az(t)+Bu_c(t)+B\Psi \left( z(t),z(t-\tau ),t\right) , \end{aligned}$$

where $z(t-\tau )$ is the delayed state vector. This representation is useful because the controller can be designed in terms of the measured current state while the delayed and nonlinear cutting-force contribution is treated as a bounded matched uncertainty. Since the uncertainty enters through the same acceleration channel as the control input, it is a matched disturbance, which is favourable for sliding mode control.

The output available for feedback is assumed to be the tool vibration displacement, velocity or acceleration measured near the spindle/tool location. In simulation, acceleration signals may be numerically integrated or filtered to estimate velocity and displacement, as in AVD-based vibration-control studies. The measurement vector may be written as

30 $$\begin{aligned} y_m(t)=C_z z(t)+v_m(t), \end{aligned}$$

where $C_z$ is the measurement matrix and $v_m(t)$ denotes bounded measurement noise. In the present modelling section, perfect state availability is assumed for clarity; however, Eq. (30) indicates how the model can later be extended to observer-based or filtered state reconstruction. This is relevant because RL policies and SMC switching terms can be sensitive to noisy velocity or sliding-surface estimates, and therefore filtering will be important in numerical and experimental implementation.

The desired chatter-free state is

31 $$\begin{aligned} z_d(t)=0, \end{aligned}$$

which corresponds to zero relative tool displacement and zero relative tool velocity around the nominal cutting equilibrium. The vibration error is therefore

32 $$\begin{aligned} e(t)=x_c(t)-x_{c,d}(t)=x_c(t), \quad \dot{e}(t)=\dot{x}_c(t). \end{aligned}$$

For the next controller-design section, the sliding surface is introduced as

33 $$\begin{aligned} s(t)=\dot{e}(t)+\Lambda _s e(t), \end{aligned}$$

where $\Lambda _s\in \mathbb {R}^{2\times 2}$ is a positive definite diagonal matrix. The sliding surface in Eq. (33) combines displacement and velocity errors and is physically meaningful for vibration suppression. Similar sliding-surface constructions are widely used in SMC vibration-control studies because they allow the designer to impose a desired first-order error dynamics once the trajectory reaches the sliding manifold. Qiu and Chen (2025) further showed that the sliding surface can be used directly as a reinforcement-learning state input for vibration control, which motivates the proposed SMC-RL architecture^11^.

### Control-input decomposition for the proposed SMC-RL framework

The total AVD control input is decomposed into a robust sliding-mode component and a reinforcement-learning compensation component:

34 $$\begin{aligned} u_c(t)=u_{SMC}(t)+u_{RL}(t). \end{aligned}$$

This structure is inspired by the combined SMC-RL control architecture of Long et al. (2021), where the sliding mode controller acts as the main controller and the actor-critic RL controller supplies an auxiliary compensation torque for vibration suppression^14^. In the present milling application, $u_{SMC}(t)$ will be designed to dominate bounded nonlinear cutting and damper-friction disturbances, while $u_{RL}(t)$ will be trained to reduce residual vibration, improve transient response and smooth the control effort.

For the mathematical model to remain compatible with Lyapunov analysis, the RL output is assumed to be bounded:

35 $$\begin{aligned} \Vert u_{RL}(t)\Vert \le \bar{u}_{RL}, \quad \forall t\ge 0, \end{aligned}$$

where $\bar{u}_{RL}$ is determined by actuator saturation or by the output layer of the RL policy. In practical implementation, this can be achieved by using a hyperbolic tangent activation function and scaling the policy output to the allowable AVD force range. The bounded-action assumption is essential because a learning controller without explicit bounds may generate unsafe actuator commands during exploration. With Eqs. (25) and (35), the subsequent controller section can establish sufficient conditions for the SMC gain such that the closed-loop milling system remains uniformly ultimately bounded.

The RL state vector can be constructed from measurable vibration and sliding-surface information:

36 $$\begin{aligned} \chi (t)=\begin{bmatrix}x(t)&y(t)&\dot{x}(t)&\dot{y}(t)&s_x(t)&s_y(t)&u_x(t-\Delta t)&u_y(t-\Delta t)\end{bmatrix}^{T}, \end{aligned}$$

where $\Delta t$ is the controller sampling interval used in the digital implementation. Alternatively, a reduced state based mainly on the sliding surface may be used:

37 $$\begin{aligned} \chi _s(t)=\begin{bmatrix}s_x(t)&s_y(t)&\dot{s}_x(t)&\dot{s}_y(t)\end{bmatrix}^{T}. \end{aligned}$$

The reduced form in Eq. (37) is motivated by Qiu and Chen (2025), who used the sliding surface as the state input to learn a robust vibration-control policy using A3C^11^. The reward function for the proposed milling controller will be selected to penalize vibration amplitude, sliding-surface magnitude, control energy and control variation. A typical reward form is

38 $$\begin{aligned} r(t)=-\left( w_1\Vert x_c(t)\Vert ^2+w_2\Vert \dot{x}_c(t)\Vert ^2+w_3\Vert s(t)\Vert ^2+w_4\Vert u_c(t)\Vert ^2+w_5\Vert \dot{u}_c(t)\Vert ^2\right) , \end{aligned}$$

where $w_1,\ldots ,w_5$ are non-negative weighting factors. Equation (38) encourages the agent to reduce chatter amplitude while avoiding unnecessarily large or rapidly varying control actions. The final SMC-RL design and Lyapunov boundedness theorem will be developed using the model established in Eqs. (15)–(38).

## Sliding mode control with reinforcement learning for active chatter mitigation

This section presents the proposed active chatter mitigation strategy based on the integration of continuous-time sliding mode control (SMC) and reinforcement learning (RL). The control objective is to suppress the self-excited vibration of the milling tool in the *x*- and *y*-directions by generating an active control force through the active vibration damper (AVD). Sliding mode control is selected as the main robust controller because of its well-established ability to handle nonlinear systems, parameter variations and bounded disturbances through a discontinuous switching action^21,22^. However, conventional SMC may produce high-frequency oscillations in the control input, commonly referred to as chattering, especially when a large switching gain is selected to dominate uncertainty^9,10^.

To reduce this drawback, the present work combines SMC with an RL-based auxiliary compensation mechanism. The role of the SMC component is to guarantee robust convergence toward the sliding surface, whereas the RL component learns a bounded compensation signal from the observed vibration states and sliding variable. This idea is consistent with recent developments in intelligent vibration control, where actor-critic RL has been integrated with SMC to improve adaptability and suppress residual vibration in flexible mechanical systems^11,14^. The use of actor-critic learning is suitable for this application because the AVD control force is continuous and the milling process contains nonlinear and uncertain dynamics. Actor-critic and policy-gradient methods have been widely used for continuous control tasks because they allow the policy to be directly parameterised and updated according to the expected return^13,23–25^.

**Fig. 2:**
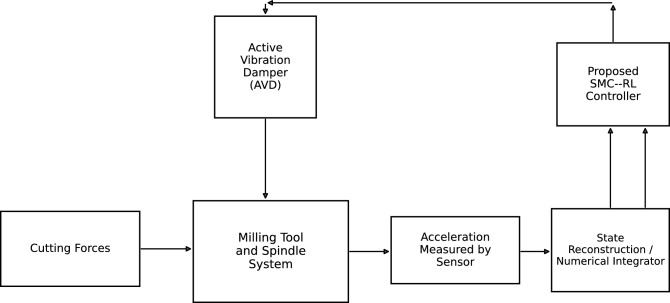
Overview of the proposed active chatter-control process using the active vibration damper (AVD) and the SMC-RL controller. Cutting forces excite the milling tool and spindle system. The measured acceleration signal is processed through state reconstruction/numerical integration and then supplied to the proposed SMC-RL control algorithm, which generates the control force for the AVD to suppress chatter vibration.

The active milling system derived in the mathematical modelling section can be expressed in a compact nonlinear state-space form as

39 $$\begin{aligned} \dot{z}(t)=Az(t)+B u_c(t)+\Phi (z,t), \end{aligned}$$

where

40 $$\begin{aligned} z(t)= \begin{bmatrix} x(t)&y(t)&\dot{x}(t)&\dot{y}(t) \end{bmatrix}^{T} \end{aligned}$$

is the state vector, *A* is the nominal state matrix, *B* is the input matrix associated with the AVD control force, $u_c(t)=[u_{cx}(t)\;u_{cy}(t)]^T$ is the active control input, and $\Phi (z,t)$ represents the lumped nonlinear and uncertain dynamics. In the present milling application, $\Phi (z,t)$ includes the nonlinear regenerative cutting forces, the damper-friction nonlinearities, unmodelled dynamics, parameter variations and external disturbances (Fig. 2). The use of a lumped uncertainty representation is standard in SMC design because the exact nonlinear dynamics do not need to be fully known, provided that their total effect is bounded^9,21,22^.

### Assumption 1

The lumped nonlinear uncertainty $\Phi (z,t)$ is bounded and satisfies

41 $$\begin{aligned} \Vert \Phi (z,t)\Vert \le \bar{\Phi }, \end{aligned}$$

where $\bar{\Phi }>0$ is an unknown but finite constant. This assumption is physically reasonable for the milling process because the cutting forces, damper friction and tool vibration response remain finite under admissible machining conditions and actuator limitations^4^.

The objective of the proposed controller is to minimise the regenerative chatter vibration of the milling tool. Since chatter suppression requires the tool vibration to be driven toward the equilibrium position, the desired displacement and velocity are selected as

42 $$\begin{aligned} x_d(t)=0,\qquad y_d(t)=0,\qquad \dot{x}_d(t)=0,\qquad \dot{y}_d(t)=0. \end{aligned}$$

The displacement error vector is defined as

43 $$\begin{aligned} e(t)=x_c(t)-x_d(t)= \begin{bmatrix} x(t)\\ y(t) \end{bmatrix}, \end{aligned}$$

where $x_c(t)=[x(t)\;y(t)]^T$ is the tool displacement vector. The corresponding velocity error is

44 $$\begin{aligned} \dot{e}(t)= \begin{bmatrix} \dot{x}(t)\\ \dot{y}(t) \end{bmatrix}. \end{aligned}$$

Therefore, reducing *e*(*t*) and $\dot{e}(t)$ directly corresponds to attenuating the chatter displacement and velocity responses in both vibration directions. The controller is designed to ensure that the vibration state remains bounded and converges to a small neighbourhood of the origin despite nonlinear cutting-force effects, damper friction and modelling uncertainty.

### Sliding surface design

The sliding surface is selected as a linear combination of the vibration displacement error and velocity error:

45 $$\begin{aligned} s(t)=\dot{e}(t)+\Lambda e(t), \end{aligned}$$

where $s(t)=[s_x(t)\;s_y(t)]^T$ is the sliding surface vector and $\Lambda$ is a positive definite matrix:

46 $$\begin{aligned} \Lambda = \begin{bmatrix} \lambda _x & 0\\ 0 & \lambda _y \end{bmatrix}, \qquad \lambda _x>0,\quad \lambda _y>0. \end{aligned}$$

The sliding variables in the two directions are therefore

47 $$\begin{aligned} \begin{aligned} s_x(t)&=\dot{x}(t)+\lambda _x x(t),\\ s_y(t)&=\dot{y}(t)+\lambda _y y(t). \end{aligned} \end{aligned}$$

When the system reaches the sliding surface, $s(t)=0$, the error dynamics become

48 $$\begin{aligned} \dot{e}(t)+\Lambda e(t)=0. \end{aligned}$$

Since $\Lambda$ is positive definite, Eq. (48) gives exponentially stable error dynamics. Thus, the chatter mitigation problem is transformed into the problem of forcing the vibration trajectory to reach the sliding surface and remain close to it. This structure follows the classical SMC principle in which the closed-loop performance is shaped by an appropriately designed sliding manifold^21,22^.

### Baseline continuous-time sliding mode controller

The baseline continuous-time SMC law is composed of two terms:

49 $$\begin{aligned} u_{\textrm{SMC}}(t)=u_{\textrm{eq}}(t)+u_{\textrm{sw}}(t), \end{aligned}$$

where $u_{\textrm{eq}}(t)$ is the equivalent control and $u_{\textrm{sw}}(t)$ is the switching control. The equivalent control compensates the nominal dynamics of the milling system, while the switching term provides robustness against bounded uncertainty and disturbances.

For the two-degree-of-freedom milling vibration model, the equivalent control can be expressed as

50 $$\begin{aligned} u_{\textrm{eq}}(t)=M_m\left[ -\Lambda \dot{e}(t)\right] +C_m\dot{x}_c(t)+K_m x_c(t)-F_m(t)+d_c(t), \end{aligned}$$

where $M_m$, $C_m$ and $K_m$ are the equivalent mass, damping and stiffness matrices, respectively, $F_m(t)$ is the cutting-force vector, and $d_c(t)$ is the damper-friction vector. In practice, the exact values of $F_m(t)$ and $d_c(t)$ are not completely available because they contain nonlinear and uncertain components. Therefore, the implementable equivalent control is written using nominal estimates:

51 $$\begin{aligned} \hat{u}_{\textrm{eq}}(t)=M_m\left[ -\Lambda \dot{e}(t)\right] +C_m\dot{x}_c(t)+K_m x_c(t)-\hat{F}_m(t)+\hat{d}_c(t), \end{aligned}$$

where $\hat{F}_m(t)$ and $\hat{d}_c(t)$ are the nominal estimates of the cutting-force and damper-friction terms.

The conventional robust switching term is given by

52 $$\begin{aligned} u_{\textrm{sw}}(t)=-K_s\operatorname {sgn}\left( s(t)\right) , \end{aligned}$$

where

53 $$\begin{aligned} K_s= \begin{bmatrix} k_{sx} & 0\\ 0 & k_{sy} \end{bmatrix}, \qquad k_{sx}>0,\quad k_{sy}>0 \end{aligned}$$

is the switching-gain matrix. The sign function is applied component-wise:

54 $$\begin{aligned} \operatorname {sgn}(s)= \begin{bmatrix} \operatorname {sgn}(s_x)\\ \operatorname {sgn}(s_y) \end{bmatrix}. \end{aligned}$$

Although Eq. (52) can provide strong robustness, it may produce high-frequency switching in the control force. This chattering phenomenon is one of the main practical limitations of SMC and can be harmful in active vibration control because it may excite unmodelled actuator dynamics and increase mechanical wear^9,10^. To reduce this effect, the discontinuous sign function is replaced by a saturation function:

55 $$\begin{aligned} u_{\textrm{sw}}(t)=-K_s\operatorname {sat}\left( \frac{s(t)}{\varphi }\right) , \end{aligned}$$

where $\varphi =[\varphi _x\;\varphi _y]^T$ is the boundary-layer vector. The saturation function is defined as

56 $$\begin{aligned} \operatorname {sat}\left( \frac{s_i}{\varphi _i}\right) = {\left\{ \begin{array}{ll} 1, & s_i>\varphi _i,\\ \dfrac{s_i}{\varphi _i}, & |s_i|\le \varphi _i,\\ -1, & s_i<-\varphi _i, \end{array}\right. } \qquad i=x,y. \end{aligned}$$

The baseline SMC law becomes

57 $$\begin{aligned} u_{\textrm{SMC}}(t)=\hat{u}_{\textrm{eq}}(t)-K_s\operatorname {sat}\left( \frac{s(t)}{\varphi }\right) . \end{aligned}$$

The saturation function reduces control discontinuity near the sliding surface. However, the boundary layer also introduces a residual region around the ideal sliding surface, meaning that the vibration states converge to a small neighbourhood rather than exactly to the origin^9,21^.

### Reinforcement learning-assisted compensation

The performance of the baseline SMC depends strongly on the values of $K_s$ and $\varphi$. A large switching gain improves robustness but may increase control chattering, whereas a small switching gain may be insufficient to compensate the nonlinear cutting force, damper friction and modelling errors. To address this trade-off, an RL-based compensation controller is introduced. The RL component learns a bounded auxiliary control signal from the observed vibration states and the sliding surface.

The total active control force applied by the AVD is defined as

58 $$\begin{aligned} u_c(t)=u_{\textrm{SMC}}(t)+u_{\textrm{RL}}(t), \end{aligned}$$

where

59 $$\begin{aligned} u_{\textrm{RL}}(t)= \begin{bmatrix} u_{\textrm{RL},x}(t)\\ u_{\textrm{RL},y}(t) \end{bmatrix} \end{aligned}$$

is the RL compensation signal. This combined structure follows the philosophy of using model-based robust control as the main stabilising mechanism and learning-based compensation as an adaptive auxiliary input^11,14^. Similar ideas have been used in flexible robotic and structural vibration control, where RL improves the adaptability of the controller in the presence of nonlinear, coupled and uncertain dynamics^13,23,24^.

To keep the learning-based compensation compatible with the stability analysis, the RL output is bounded using a hyperbolic tangent function:

60 $$\begin{aligned} u_{\textrm{RL}}(t)=u_{\max }\tanh \left( \pi _{\theta }\left( \chi (t)\right) \right) , \end{aligned}$$

where $u_{\max }>0$ is the maximum admissible RL compensation force, $\pi _{\theta }(\cdot )$ is the actor policy parameterised by $\theta$, and $\chi (t)$ is the RL state vector. The bounded output condition is expressed as

61 $$\begin{aligned} \Vert u_{\textrm{RL}}(t)\Vert \le u_{\max }. \end{aligned}$$

This condition prevents the RL controller from generating excessive control force and is essential for maintaining a stability-oriented control structure.

### Relationship between SMC and RL and the reduced SMC case

The proposed control structure is intentionally formulated as a hierarchical robust-learning architecture rather than as a purely learning-based controller. In this structure, SMC acts as the primary stabilising controller, while RL acts as a bounded auxiliary compensation mechanism. The role of SMC is to impose the sliding dynamics, dominate matched bounded uncertainty and provide the Lyapunov-based boundedness guarantee. The role of RL is not to replace this robust control mechanism, but to improve the transient and residual vibration response by learning compensation from the observed vibration states and sliding variables. This interpretation is consistent with robust learning-assisted vibration-control structures in which a model-based robust controller is used as the main stabilising component and a learning-based term is added only as an auxiliary compensation signal^11,14^.

The relationship between the two components can be expressed by decomposing the total AVD control input into the SMC component and the RL compensation component:

62 $$\begin{aligned} u_c(t)=u_{\textrm{SMC}}(t)+u_{\textrm{RL}}(t). \end{aligned}$$

Here, $u_{\textrm{SMC}}(t)$ is the robust sliding-mode control force and $u_{\textrm{RL}}(t)$ is the bounded learning-based compensation force. Substituting the SMC law into the total control input gives

63 $$\begin{aligned} u_c(t)=\hat{u}_{\textrm{eq}}(t)-K_s\,\operatorname {sat}\left( \frac{s(t)}{\varphi }\right) +u_{\textrm{RL}}(t). \end{aligned}$$

Thus, the SMC part determines the reaching behaviour toward the sliding surface, whereas the RL part modifies the input within a bounded range to improve attenuation performance. Since the RL output is constrained by

64 $$\begin{aligned} \left| u_{\textrm{RL}}(t)\right| \le u_{\max }, \end{aligned}$$

the learning component cannot generate an unbounded force or override the stabilising structure imposed by SMC.

If the RL component is removed by setting

65 $$\begin{aligned} u_{\textrm{RL}}(t)=0, \end{aligned}$$

the proposed controller reduces to the standard SMC law:

66 $$\begin{aligned} \begin{aligned} u_c(t)&= u_{\textrm{SMC}}(t) \ =\hat{u}_{\textrm{eq}}(t) {}-{} K_s\operatorname {sat}\left( \frac{s(t)}{\varphi }\right) . \end{aligned} \end{aligned}$$

In this reduced case, the boundedness result remains valid under the usual SMC gain condition. Specifically, if the lumped uncertainty satisfies

67 $$\begin{aligned} \left| \Delta (t)\right| \le \bar{\Delta }, \end{aligned}$$

then the reduced SMC controller satisfies the Lyapunov reaching condition when

68 $$\begin{aligned} \lambda _{\min }(K_s)>\bar{\Delta }. \end{aligned}$$

Therefore, the stability or boundedness satisfaction does not depend on the presence of RL. The SMC component alone can guarantee boundedness if the switching gain is selected sufficiently large to dominate the bounded uncertainty^10,21,22^.

However, eliminating RL also removes the adaptive compensation capability. The standard SMC controller must then rely only on a fixed switching gain and boundary layer to balance robustness and chattering reduction. A larger switching gain improves uncertainty rejection but may increase high-frequency control activity, whereas a smaller gain may reduce chattering but leave residual vibration. This trade-off is particularly relevant in milling chatter control because the nonlinear cutting force, regenerative delay and damper friction may vary with cutting conditions. The RL component is therefore introduced to improve performance within the stable SMC framework by learning a bounded auxiliary force that reduces residual chatter and smooths the control behaviour.

Accordingly, the role of RL in the proposed framework is deliberately limited but important. It is limited because it is not used as the main stability-guaranteeing controller; this avoids relying on an unconstrained black-box learning policy for closed-loop stability. It is important because it improves the performance of the robust SMC baseline by compensating residual nonlinear effects and reducing the need for overly conservative switching action. Thus, SMC provides the stability backbone, while RL improves attenuation performance and control smoothness.

### RL state, action and reward design

The RL state vector must include information that reflects the current vibration condition and the convergence behaviour of the sliding-mode controller. In the proposed structure, the RL state is selected as

69 $$\begin{aligned} \chi (t)= \begin{bmatrix} x(t)&y(t)&\dot{x}(t)&\dot{y}(t)&s_x(t)&s_y(t)&u_c(t-\Delta t) \end{bmatrix}^{T}, \end{aligned}$$

where $\Delta t$ is the RL sampling interval. The displacement and velocity terms describe the instantaneous chatter response, while the sliding variables $s_x(t)$ and $s_y(t)$ provide information about the distance from the sliding surface. The previous control input $u_c(t-\Delta t)$ is included to discourage abrupt control variation. The use of the sliding surface as part of the RL state is motivated by sliding mode reinforcement learning, where the sliding variable provides a compact and control-relevant representation of the system error dynamics^11^.

The RL action is defined as the auxiliary compensation force:

70 $$\begin{aligned} a(t)=u_{\textrm{RL}}(t). \end{aligned}$$

For the two-axis milling system, this gives

71 $$\begin{aligned} a(t)= \begin{bmatrix} u_{\textrm{RL},x}(t)\\ u_{\textrm{RL},y}(t) \end{bmatrix}. \end{aligned}$$

The reward function is designed to penalise vibration amplitude, velocity response, deviation from the sliding surface, control effort and high-frequency changes in the control signal. The instantaneous reward is given by

72 $$\begin{aligned} r(t)=- \left[ w_1\Vert x_c(t)\Vert ^2+ w_2\Vert \dot{x}_c(t)\Vert ^2+ w_3\Vert s(t)\Vert ^2+ w_4\Vert u_c(t)\Vert ^2+ w_5\Vert \Delta u_c(t)\Vert ^2 \right] , \end{aligned}$$

where $w_1$, $w_2$, $w_3$, $w_4$ and $w_5$ are positive weighting coefficients, and

73 $$\begin{aligned} \Delta u_c(t)=u_c(t)-u_c(t-\Delta t). \end{aligned}$$

The first and second terms in Eq. (72) promote chatter suppression. The third term encourages convergence toward the sliding surface. The fourth term limits the actuator effort, while the fifth term reduces aggressive variations in the AVD force. Therefore, maximising the cumulative reward encourages both vibration attenuation and smoother control behaviour.

### Actor-critic learning structure and convergence of the training law

An actor-critic reinforcement learning structure is adopted because the chatter-control problem involves continuous vibration states and continuous AVD control forces. In this framework, the actor generates the auxiliary compensation force, while the critic evaluates the quality of the selected action by estimating the value of the current state. This structure is suitable for the proposed controller because the SMC component provides the robust stabilising action, whereas the actor-critic component learns a bounded compensation signal that improves residual vibration attenuation. Actor-critic and policy-gradient methods are widely used in continuous-control problems because they update the control policy directly using value-function information^13,23–25^.

The actor policy is expressed as

74 $$\begin{aligned} u_{\textrm{RL}}(t)=\pi _{\theta }\left( \chi (t)\right) . \end{aligned}$$

where $\theta$ denotes the actor parameters and $\chi (t)$ is the RL state vector. In the present implementation, the actor output is passed through a bounded activation function so that

75 $$\begin{aligned} u_{\textrm{RL}}(t)=u_{\max }\tanh \left( \pi _{\theta }\left( \chi (t)\right) \right) . \end{aligned}$$

This bounded output is important because it prevents the RL component from generating excessive actuator force and keeps the learning-based compensation compatible with the Lyapunov boundedness analysis.

The critic approximates the state-value function:

76 $$\begin{aligned} V_{\omega }\left( \chi (t)\right) \approx \mathbb {E}\left[ \sum _{j=0}^{\infty }\gamma ^{j}r(t+j\Delta t)\right] . \end{aligned}$$

where $\omega$ denotes the critic parameters and $\gamma \in (0,1)$ is the discount factor. The temporal-difference error is computed as

77 $$\begin{aligned} \delta _{\textrm{TD}}(t)=r(t)+\gamma V_{\omega }\left( \chi (t+\Delta t)\right) -V_{\omega }\left( \chi (t)\right) . \end{aligned}$$

The temporal-difference error measures the inconsistency between the current value estimate and the one-step reward-plus-future-value estimate. Therefore, reducing $\delta _{\textrm{TD}}(t)$ improves the critic approximation and provides a more reliable learning signal for the actor.

The critic loss function is defined as

78 $$\begin{aligned} L_c(\omega )=\frac{1}{2}\delta _{\textrm{TD}}^2(t). \end{aligned}$$

The critic parameters are updated by gradient descent:

79 $$\begin{aligned} \omega (t+\Delta t)=\omega (t)-\alpha _c\nabla _{\omega }L_c(\omega ). \end{aligned}$$

where $\alpha _c>0$ is the critic learning rate. Since

80 $$\begin{aligned} \nabla _{\omega }L_c(\omega )=\delta _{\textrm{TD}}(t)\nabla _{\omega }\delta _{\textrm{TD}}(t). \end{aligned}$$

the critic update can be interpreted as a parameter adjustment that reduces the squared temporal-difference error. In practical implementation, convergence of the critic training is indicated by a decreasing and bounded critic loss:

81 $$\begin{aligned} L_c\left( \omega (t+\Delta t)\right) \le L_c\left( \omega (t)\right) . \end{aligned}$$

After sufficient training iterations, the critic loss is expected to decrease and settle to a small bounded neighbourhood because stochastic approximation and function-approximation errors may prevent exact zero error.

The actor is updated using the temporal-difference error as an estimate of the advantage of the selected action. The actor objective is to maximise the expected discounted return:

82 $$\begin{aligned} J_a(\theta )=\mathbb {E}\left[ \sum _{j=0}^{\infty }\gamma ^{j}r(t+j\Delta t)\right] . \end{aligned}$$

Using the policy-gradient form, the actor parameters are updated as

83 $$\begin{aligned} \theta (t+\Delta t)=\theta (t)+\alpha _a\delta _{\textrm{TD}}(t)\nabla _{\theta }\log \pi _{\theta }\left( u_{\textrm{RL}}(t)\mid \chi (t)\right) . \end{aligned}$$

where $\alpha _a>0$ is the actor learning rate. This update increases the probability of compensation actions that produce positive temporal-difference improvement and decreases the probability of actions that reduce the expected return. Thus, the actor does not learn independently of the critic; rather, the critic supplies the TD-error signal that directs the actor toward actions that reduce vibration, reduce sliding-surface deviation and limit unnecessary control effort.

For the present milling chatter problem, convergence of the actor-critic training law is interpreted in a practical closed-loop control sense rather than as exact global optimality of a nonlinear neural-network policy. The policy is considered to have converged when the following conditions are simultaneously observed during offline training:

84 $$\begin{aligned} \left| \delta _{\textrm{TD}}(t)\right| \rightarrow \varepsilon _{\delta }. \end{aligned}$$

85 $$\begin{aligned} L_c(\omega )\rightarrow \varepsilon _c. \end{aligned}$$

86 $$\begin{aligned} \left| J_a(\theta (t+\Delta t))-J_a(\theta (t))\right| \rightarrow \varepsilon _J. \end{aligned}$$

87 $$\begin{aligned} \left| u_{\textrm{RL}}(t)\right| \le u_{\max }. \end{aligned}$$

where $\varepsilon _{\delta }$, $\varepsilon _c$ and $\varepsilon _J$ are small positive constants. These conditions indicate that the critic prediction error has become bounded, the critic loss has settled, the improvement in expected return has become small, and the learned actor remains within the admissible actuator force limit.

In the proposed framework, the actor-critic training is performed offline using the nonlinear milling simulation model before final closed-loop evaluation. The policy is trained by repeatedly applying the bounded RL compensation force to the simulated milling environment, observing the vibration response, computing the reward, updating the critic through Eq. (79), and updating the actor through Eq. (83). After the training reward converges and the critic loss becomes bounded, the trained actor is fixed and used as the RL compensation element in the SMC–RL controller. Therefore, the closed-loop controller used in the final simulation does not rely on unsafe online exploration during machining.

The convergence of the training law is also connected to the stability-oriented structure of the proposed controller. The actor-critic algorithm improves the auxiliary compensation term, but the SMC component remains responsible for the robust boundedness guarantee. Even if the actor-critic training reaches only a bounded local policy rather than a globally optimal policy, the RL output is saturated and therefore remains bounded. Consequently, the learning component improves the vibration attenuation performance without invalidating the boundedness condition established for the SMC–RL closed-loop system.

### Complete SMC-RL control law

By combining the baseline SMC law and the bounded RL compensation signal, the complete active control law becomes

88 $$\begin{aligned} u_c(t)= \hat{u}_{\textrm{eq}}(t) - K_s\operatorname {sat}\left( \frac{s(t)}{\varphi }\right) + u_{\max }\tanh \left( \pi _{\theta }\left( \chi (t)\right) \right) . \end{aligned}$$

The first term compensates the nominal milling dynamics. The second term provides robust sliding-mode action against bounded uncertainty. The third term supplies adaptive RL-based compensation for residual nonlinear effects. The proposed controller therefore combines model-based robustness with data-driven adaptability.

The implementation procedure is summarised as follows:

1. Measure or estimate the tool displacement and velocity responses *x*(*t*), *y*(*t*), $\dot{x}(t)$ and $\dot{y}(t)$.
2. Compute the vibration error vector *e*(*t*) using Eq. (43).
3. Compute the sliding surface *s*(*t*) using Eq. (45).
4. Compute the nominal equivalent control $\hat{u}_{\textrm{eq}}(t)$ using Eq. (51).
5. Generate the robust SMC term using Eq. (55).
6. Construct the RL state vector $\chi (t)$ using Eq. (69).
7. Generate the bounded RL compensation signal $u_{\textrm{RL}}(t)$ using Eq. (60).
8. Apply the total AVD control force $u_c(t)$ using Eq. (88).
9. Evaluate the reward function in Eq. (72) and update the actor and critic networks using Eqs. (79) and (83).

### Boundedness analysis of the proposed SMC-RL controller

This subsection presents a Lyapunov-based boundedness analysis of the proposed SMC-RL controller. The purpose of the analysis is to show that the sliding surface, vibration error and milling-tool vibration states remain bounded when the active vibration damper is controlled by the combined SMC-RL law. The proof follows the standard robust sliding-mode stability framework, where the sliding-mode component is responsible for guaranteeing robustness against bounded uncertainty, while the reinforcement learning component is treated as a bounded auxiliary compensation input^10,21,22^. This bounded-output treatment is important because the RL policy is not used as an unconstrained black-box controller; rather, its output is restricted by the saturation structure introduced in Eq. (60).

Starting from the active milling dynamics,

89 $$\begin{aligned} M_m\ddot{x}_c(t)+C_m\dot{x}_c(t)+K_mx_c(t) = F_m(t)+u_c(t)-d_c(t), \end{aligned}$$

where $x_c(t)=[x(t)\;y(t)]^T$, the vibration error is defined as

90 $$\begin{aligned} e(t)=x_c(t)-x_d(t). \end{aligned}$$

For chatter suppression, the desired vibration is zero, i.e.,

91 $$\begin{aligned} x_d(t)=0,\qquad \dot{x}_d(t)=0. \end{aligned}$$

Therefore,

92 $$\begin{aligned} e(t)=x_c(t),\qquad \dot{e}(t)=\dot{x}_c(t),\qquad \ddot{e}(t)=\ddot{x}_c(t). \end{aligned}$$

The sliding surface is selected as

93 $$\begin{aligned} s(t)=\dot{e}(t)+\Lambda e(t), \end{aligned}$$

where $\Lambda =\Lambda ^T>0$. Differentiating Eq. (93) gives

94 $$\begin{aligned} \dot{s}(t)=\ddot{e}(t)+\Lambda \dot{e}(t). \end{aligned}$$

Multiplying Eq. (94) by $M_m$ and using Eq. (92) gives

95 $$\begin{aligned} M_m\dot{s}(t) = M_m\ddot{x}_c(t)+M_m\Lambda \dot{x}_c(t). \end{aligned}$$

From Eq. (89),

96 $$\begin{aligned} M_m\ddot{x}_c(t) = -C_m\dot{x}_c(t)-K_mx_c(t)+F_m(t)+u_c(t)-d_c(t). \end{aligned}$$

Substituting Eq. (96) into Eq. (95) yields

97 $$\begin{aligned} M_m\dot{s}(t) = -C_m\dot{x}_c(t)-K_mx_c(t)+F_m(t)-d_c(t) +u_c(t)+M_m\Lambda \dot{x}_c(t). \end{aligned}$$

The proposed control law is written as

98 $$\begin{aligned} u_c(t) = \hat{u}_{\textrm{eq}}(t) - K_s\operatorname {sat}\left( \frac{s(t)}{\varphi }\right) + u_{\textrm{RL}}(t), \end{aligned}$$

where $K_s=K_s^T>0$ is the switching-gain matrix, $\varphi =[\varphi _x\;\varphi _y]^T$ is the boundary-layer vector and $u_{\textrm{RL}}(t)$ is the bounded reinforcement-learning compensation signal. The nominal equivalent control is selected as

99 $$\begin{aligned} \hat{u}_{\textrm{eq}}(t) = C_m\dot{x}_c(t)+K_mx_c(t) -M_m\Lambda \dot{x}_c(t) -\hat{F}_m(t)+\hat{d}_c(t), \end{aligned}$$

where $\hat{F}_m(t)$ and $\hat{d}_c(t)$ are the nominal estimates of the cutting-force and damper-friction terms, respectively.

Substituting Eqs. (98) and (99) into Eq. (97) gives

100 $$\begin{aligned} M_m\dot{s}(t) = \Delta (t) - K_s\operatorname {sat}\left( \frac{s(t)}{\varphi }\right) + u_{\textrm{RL}}(t), \end{aligned}$$

where the lumped modelling uncertainty is

101 $$\begin{aligned} \Delta (t) = F_m(t)-\hat{F}_m(t)+\hat{d}_c(t)-d_c(t). \end{aligned}$$

The term $\Delta (t)$ contains the cutting-force modelling error, the damper-friction modelling error, unmodelled nonlinearities and bounded disturbances. The following assumptions are introduced for the stability analysis.

#### Assumption 1

The mass matrix $M_m$ is symmetric and positive definite:

102 $$\begin{aligned} M_m=M_m^T>0. \end{aligned}$$

Therefore, there exist positive constants $\lambda _{\min }(M_m)$ and $\lambda _{\max }(M_m)$ such that

103 $$\begin{aligned} \lambda _{\min }(M_m)\Vert s(t)\Vert ^2 \le s^T(t)M_m s(t) \le \lambda _{\max }(M_m)\Vert s(t)\Vert ^2. \end{aligned}$$

#### Assumption 2

The lumped uncertainty $\Delta (t)$ is bounded:

104 $$\begin{aligned} \Vert \Delta (t)\Vert \le \bar{\Delta }, \end{aligned}$$

where $\bar{\Delta }>0$ is a finite constant. This assumption is physically reasonable because the cutting forces, friction forces and external disturbances remain finite under admissible cutting conditions and actuator constraints^3,4^.

#### Assumption 3

The reinforcement-learning compensation signal is bounded:

105 $$\begin{aligned} \Vert u_{\textrm{RL}}(t)\Vert \le u_{\max }, \end{aligned}$$

where $u_{\max }>0$ is the maximum admissible RL compensation force. This is ensured by defining the RL output as

106 $$\begin{aligned} u_{\textrm{RL}}(t)=u_{\max }\tanh \left( \pi _{\theta }(\chi (t))\right) . \end{aligned}$$

Hence, the RL policy may adapt the auxiliary compensation, but it cannot generate an unbounded active force.

#### Theorem 1

Consider the nonlinear active milling system in Eq. (89) controlled by the proposed SMC-RL law in Eq. (98). Suppose that Assumptions 1–3 hold. If the switching-gain matrix $K_s$ is selected such that

107 $$\begin{aligned} \lambda _{\min }(K_s)>\bar{\Delta }+u_{\max }, \end{aligned}$$

then the sliding surface *s*(*t*) is driven to a bounded neighbourhood of the origin. Consequently, the vibration error *e*(*t*), velocity error $\dot{e}(t)$ and state vector $z(t)=[x(t)\;y(t)\;\dot{x}(t)\;\dot{y}(t)]^T$ are uniformly ultimately bounded.

#### Proof

Choose the Lyapunov candidate function

108 $$\begin{aligned} V(t)=\frac{1}{2}s^T(t)M_m s(t). \end{aligned}$$

Since $M_m=M_m^T>0$, the Lyapunov function is positive definite. Using Eq. (103), it satisfies

109 $$\begin{aligned} \frac{1}{2}\lambda _{\min }(M_m)\Vert s(t)\Vert ^2 \le V(t) \le \frac{1}{2}\lambda _{\max }(M_m)\Vert s(t)\Vert ^2. \end{aligned}$$

Taking the derivative of Eq. (108) gives

110 $$\begin{aligned} \dot{V}(t) = \frac{1}{2}\dot{s}^T(t)M_m s(t) + \frac{1}{2}s^T(t)M_m\dot{s}(t). \end{aligned}$$

Since $M_m=M_m^T$, Eq. (110) becomes

111 $$\begin{aligned} \dot{V}(t) = s^T(t)M_m\dot{s}(t). \end{aligned}$$

Using the sliding dynamics in Eq. (100),

112 $$\begin{aligned} \dot{V}(t) = s^T(t) \left[ \Delta (t) - K_s\operatorname {sat}\left( \frac{s(t)}{\varphi }\right) + u_{\textrm{RL}}(t) \right] . \end{aligned}$$

Expanding the right-hand side gives

113 $$\begin{aligned} \dot{V}(t) = s^T(t)\Delta (t) - s^T(t)K_s\operatorname {sat}\left( \frac{s(t)}{\varphi }\right) + s^T(t)u_{\textrm{RL}}(t). \end{aligned}$$

First, using the Cauchy-Schwarz inequality and Assumption 2,

114 $$\begin{aligned} s^T(t)\Delta (t) \le \Vert s(t)\Vert \Vert \Delta (t)\Vert \le \bar{\Delta }\Vert s(t)\Vert . \end{aligned}$$

Second, using Assumption 3,

115 $$\begin{aligned} s^T(t)u_{\textrm{RL}}(t) \le \Vert s(t)\Vert \Vert u_{\textrm{RL}}(t)\Vert \le u_{\max }\Vert s(t)\Vert . \end{aligned}$$

Third, for the switching term, outside the boundary layer the saturation function becomes the sign function:

116 $$\begin{aligned} \operatorname {sat}\left( \frac{s(t)}{\varphi }\right) = \operatorname {sgn}(s(t)). \end{aligned}$$

Therefore,

117 $$\begin{aligned} s^T(t)K_s\operatorname {sgn}(s(t)) = \sum _{i=x,y} k_{si}|s_i(t)|. \end{aligned}$$

Let

118 $$\begin{aligned} k_{\min }=\lambda _{\min }(K_s)=\min (k_{sx},k_{sy}). \end{aligned}$$

Then,

119 $$\begin{aligned} \sum _{i=x,y} k_{si}|s_i(t)| \ge k_{\min }\sum _{i=x,y}|s_i(t)|. \end{aligned}$$

Since $\Vert s(t)\Vert _1\ge \Vert s(t)\Vert _2$, it follows that

120 $$\begin{aligned} s^T(t)K_s\operatorname {sgn}(s(t)) \ge k_{\min }\Vert s(t)\Vert . \end{aligned}$$

Using Eqs. (114), (115) and (120) in Eq. (113), the Lyapunov derivative satisfies

121 $$\begin{aligned} \dot{V}(t) \le \bar{\Delta }\Vert s(t)\Vert - k_{\min }\Vert s(t)\Vert + u_{\max }\Vert s(t)\Vert . \end{aligned}$$

Hence,

122 $$\begin{aligned} \dot{V}(t) \le - \left( k_{\min }-\bar{\Delta }-u_{\max } \right) \Vert s(t)\Vert . \end{aligned}$$

Define the positive robustness margin

123 $$\begin{aligned} \eta _s = k_{\min }-\bar{\Delta }-u_{\max }. \end{aligned}$$

If the gain condition in Eq. (107) is satisfied, then $\eta _s>0$, and therefore

124 $$\begin{aligned} \dot{V}(t) \le -\eta _s\Vert s(t)\Vert . \end{aligned}$$

Using the upper bound in Eq. (109),

125 $$\begin{aligned} \Vert s(t)\Vert \ge \sqrt{\frac{2V(t)}{\lambda _{\max }(M_m)}}. \end{aligned}$$

Substituting Eq. (125) into Eq. (124) gives

126 $$\begin{aligned} \dot{V}(t) \le -\eta _s \sqrt{\frac{2}{\lambda _{\max }(M_m)}} V^{1/2}(t). \end{aligned}$$

Let

127 $$\begin{aligned} c_s= \eta _s \sqrt{\frac{2}{\lambda _{\max }(M_m)}}. \end{aligned}$$

Then

128 $$\begin{aligned} \dot{V}(t)\le -c_sV^{1/2}(t). \end{aligned}$$

For $$V(t)>0$$,

129 $$\begin{aligned} \frac{d}{dt}\sqrt{V(t)} = \frac{\dot{V}(t)}{2\sqrt{V(t)}} \le -\frac{c_s}{2}. \end{aligned}$$

Integrating Eq. (129) from 0 to *t* gives

130 $$\begin{aligned} \sqrt{V(t)} \le \sqrt{V(0)}-\frac{c_s}{2}t. \end{aligned}$$

Therefore, the sliding surface reaches the neighbourhood of the sliding manifold in finite time. The reaching time satisfies

131 $$\begin{aligned} t_r \le \frac{2\sqrt{V(0)}}{c_s} = \frac{\sqrt{2\lambda _{\max }(M_m)V(0)}}{\eta _s}. \end{aligned}$$

For the implemented controller, the discontinuous sign function is replaced by the saturation function. Therefore, exact sliding is replaced by convergence to a boundary layer around the ideal sliding surface. Define the boundary-layer set as

132 $$\begin{aligned} \Omega _{\varphi } = \left\{ s(t): |s_x(t)|\le \varphi _x,\; |s_y(t)|\le \varphi _y \right\} . \end{aligned}$$

Inside this set,

133 $$\begin{aligned} \Vert s(t)\Vert \le \Vert \varphi \Vert = \sqrt{\varphi _x^2+\varphi _y^2}. \end{aligned}$$

Thus, after the reaching phase, the sliding variable is uniformly ultimately bounded as

134 $$\begin{aligned} \limsup _{t\rightarrow \infty }\Vert s(t)\Vert \le \Vert \varphi \Vert . \end{aligned}$$

Using Eq. (109), the corresponding ultimate bound on the Lyapunov function is

135 $$\begin{aligned} \limsup _{t\rightarrow \infty }V(t) \le \frac{1}{2}\lambda _{\max }(M_m)\Vert \varphi \Vert ^2. \end{aligned}$$

It remains to show that boundedness of *s*(*t*) implies boundedness of the vibration error and the full state. From the sliding surface definition,

136 $$\begin{aligned} \dot{e}(t) = -\Lambda e(t)+s(t). \end{aligned}$$

The solution of Eq. (136) from a time instant $t_0$ is

137 $$\begin{aligned} e(t) = e^{-\Lambda (t-t_0)}e(t_0) + \int _{t_0}^{t} e^{-\Lambda (t-\tau )}s(\tau )\,d\tau . \end{aligned}$$

Since $$\Lambda = \Lambda T > 0,$$

138 $$\begin{aligned} \left\| e^{-\Lambda (t-t_0)}\right\| \le e^{-\lambda _{\min }(\Lambda )(t-t_0)}. \end{aligned}$$

Taking the norm of Eq. (137) and applying Eq. (134) gives

139 $$\begin{aligned} \Vert e(t)\Vert \le e^{-\lambda _{\min }(\Lambda )(t-t_0)}\Vert e(t_0)\Vert + \int _{t_0}^{t} e^{-\lambda _{\min }(\Lambda )(t-\tau )} \Vert s(\tau )\Vert \,d\tau . \end{aligned}$$

After the reaching phase, $\Vert s(\tau )\Vert \le \Vert \varphi \Vert$, and therefore

140 $$\begin{aligned} \Vert e(t)\Vert \le e^{-\lambda _{\min }(\Lambda )(t-t_0)}\Vert e(t_0)\Vert + \Vert \varphi \Vert \int _{t_0}^{t} e^{-\lambda _{\min }(\Lambda )(t-\tau )} d\tau . \end{aligned}$$

The integral term is

141 $$\begin{aligned} \int _{t_0}^{t} e^{-\lambda _{\min }(\Lambda )(t-\tau )} d\tau = \frac{1-e^{-\lambda _{\min }(\Lambda )(t-t_0)}}{\lambda _{\min }(\Lambda )}. \end{aligned}$$

Thus,

142 $$\begin{aligned} \Vert e(t)\Vert \le e^{-\lambda _{\min }(\Lambda )(t-t_0)}\Vert e(t_0)\Vert + \frac{\Vert \varphi \Vert }{\lambda _{\min }(\Lambda )} \left[ 1-e^{-\lambda _{\min }(\Lambda )(t-t_0)} \right] . \end{aligned}$$

Taking the upper limit as $t\rightarrow \infty$ gives

143 $$\begin{aligned} \limsup _{t\rightarrow \infty }\Vert e(t)\Vert \le \frac{\Vert \varphi \Vert }{\lambda _{\min }(\Lambda )}. \end{aligned}$$

Therefore, the vibration displacement error is uniformly ultimately bounded. Since

144 $$\begin{aligned} \dot{e}(t)=s(t)-\Lambda e(t), \end{aligned}$$

the velocity error is also bounded:

145 $$\begin{aligned} \Vert \dot{e}(t)\Vert \le \Vert s(t)\Vert +\Vert \Lambda \Vert \Vert e(t)\Vert . \end{aligned}$$

Using Eqs. (134) and (143), one obtains

146 $$\begin{aligned} \limsup _{t\rightarrow \infty }\Vert \dot{e}(t)\Vert \le \Vert \varphi \Vert + \frac{\Vert \Lambda \Vert \Vert \varphi \Vert }{\lambda _{\min }(\Lambda )}. \end{aligned}$$

Because $e(t)=x_c(t)$ and $\dot{e}(t)=\dot{x}_c(t)$ for the zero-vibration reference, boundedness of *e*(*t*) and $\dot{e}(t)$ implies boundedness of the complete milling vibration state

147 $$\begin{aligned} z(t)= \begin{bmatrix} x(t)&y(t)&\dot{x}(t)&\dot{y}(t) \end{bmatrix}^{T}. \end{aligned}$$

Hence, the closed-loop SMC-RL milling system is uniformly ultimately bounded. This completes the proof. $\square$

Theorem 1 shows that the proposed controller preserves the robustness property of sliding mode control while allowing the RL component to provide adaptive compensation. The condition in Eq. (107) has a clear physical interpretation: the switching gain must dominate the combined effect of the bounded modelling uncertainty and the maximum RL compensation force. At the same time, the saturation function introduces a finite boundary layer, which avoids discontinuous high-frequency switching and produces a practically bounded residual vibration region. The RL component is useful because it can learn part of the residual nonlinear compensation from vibration data, thereby reducing the need for an excessively conservative switching gain while maintaining bounded closed-loop behaviour.

## Numerical simulation and validation

This section validates the effectiveness of the proposed sliding mode control with reinforcement learning (SMC-RL) for chatter mitigation in the milling process. The numerical study is carried out using the dynamic milling model developed in the previous section and the active vibration damper (AVD) configuration adopted from Paul and Morales-Menendez^3^. The tool and cutting parameters used for the simulation are extracted from the parameter set by Moradi et al.^4^. The nonlinear cutting-force description is also consistent with the extended nonlinear milling formulations discussed by Moradi et al.^4^. The objective of the present validation is to compare the chatter-attenuation capability of the proposed SMC-RL controller against the uncontrolled response, a conventional PID controller and a standard SMC controller.

The numerical validation is also used to clarify the connection between the theoretical boundedness result in Theorem 1 and the simulated closed-loop behaviour. Theorem 1 establishes that the milling vibration states remain uniformly ultimately bounded when the lumped uncertainty is bounded, the reinforcement-learning compensation force is bounded, and the SMC switching gain is selected to dominate the combined uncertainty and maximum RL compensation. Therefore, the simulation is not only used to compare vibration amplitudes but also to verify whether the closed-loop responses behave consistently with the boundedness condition. In particular, the tool vibration responses *x*(*t*) and *y*(*t*) should remain bounded and decay toward a small neighbourhood of the chatter-free equilibrium, the control force should remain finite, and the RL compensation should satisfy

148 $$\begin{aligned} \left| u_{\textrm{RL}}(t)\right| \le u_{\max }. \end{aligned}$$

This condition corresponds directly to the bounded-action assumption used in the theorem.

The standard SMC case is included to show what happens when the RL component is eliminated from the control framework. In this case, the controller becomes

149 $$\begin{aligned} u_c(t)=\hat{u}_{\textrm{eq}}(t)-K_s\operatorname {sat}\left( \frac{s(t)}{\varphi }\right) . \end{aligned}$$

This comparison is important because it separates the robust stabilising contribution of SMC from the adaptive compensation contribution of RL. The uncontrolled case provides the reference chatter condition, the PID controller provides a conventional industrial benchmark, the standard SMC controller verifies the baseline robust controller without learning, and the proposed SMC-RL controller evaluates the combined effect of robust sliding-mode action and bounded learning-based compensation. The simulation comparison is therefore performed using time-domain vibration responses, mean squared error, percentage attenuation, control-force behaviour and reward convergence, which together provide a direct link between Theorem 1 and the numerical performance evaluation.

### Simulation setup

The simulation interval was selected as $0.1~\textrm{s}$ to $0.6~\textrm{s}$. The milling system was treated as a two-degree-of-freedom structure vibrating in the feed and normal directions, while the AVD was assumed to be mounted on the spindle and to contribute an active control force in both directions. As in the base study, the AVD mass was taken as $5\%$ of the main vibrating device mass^3^. The number of cutter teeth was taken as $n=4$ and the spindle speed was set to $\Omega =3000~\mathrm {rev/min}$, which corresponds to the cutting condition used in the base paper for tool-vibration simulation. The nonlinear cutting-force coefficients were retained from the same parameter set in order to preserve the chatter-generating conditions.

The principal milling and structural parameters used in the numerical study are summarised in Table 1. These values were extracted from Moradi et al.^26^ and were used as the foundation for generating the tool-vibration responses and validating the developed control strategy.

**Table 1:** Dynamic and cutting parameters used in the numerical simulation.

Parameter	Value	Unit
$m_x$	20	kg
$m_y$	20	kg
$c_x$	1200	Ns/m
$c_y$	4300	Ns/m
$k_x$	$7.2\times 10^{6}$	N/m
$k_y$	$6.48\times 10^{7}$	N/m
$\xi _1$	$6700\times 10^{9}$	N/m$^{3}$
$\xi _2$	$-4900\times 10^{6}$	N/m$^{2}$
$\xi _3$	$3000\times 10^{3}$	N/m
$\xi _4$	$1700\times 10^{3}$	N/m
$\delta _1$	$13000\times 10^{9}$	N/m$^{3}$
$\delta _2$	$-7200\times 10^{6}$	N/m$^{2}$
$\delta _3$	13	N
$\delta _4$	25	N
*n*	4	–
$\Omega$	3000	rev/min

The simulation study considered four cases:

1. milling response without active control;
2. milling response with a tuned PID controller;
3. milling response with a standard SMC controller;
4. milling response with the proposed SMC-RL controller.

The uncontrolled case provides the reference chatter response, while the PID-controlled case serves as a conventional industrial benchmark. The standard SMC case isolates the contribution of the robust sliding-mode controller without learning. The proposed SMC-RL controller combines the robust sliding-mode term with a bounded RL-based auxiliary compensation signal, as developed in the previous section.

### Controller implementation

For the benchmark case, a classical PID controller was implemented in both vibration directions. The control force generated by the PID controller can be written as

150 $$\begin{aligned} u_{\textrm{PID}}(t)=K_p e(t)+K_i\int _0^t e(\tau ),d\tau +K_d\dot{e}(t), \end{aligned}$$

where $K_p$, $K_i$ and $K_d$ are the proportional, integral and derivative gains, respectively. The PID controller was tuned to achieve the best possible chatter attenuation without causing excessive oscillation in the control force. The use of PID as a benchmark is justified because it remains one of the most common industrial control strategies^27^. where $K_p$, $K_i$ and $K_d$ are the proportional, integral and derivative gain matrices, respectively. In the present simulation, independent PID gains were used in the *x*- and *y*-directions as

151 $$\begin{aligned} K_p= \begin{bmatrix} 180&0\ 0&210 \end{bmatrix}, \qquad K_i= \begin{bmatrix} 35&0\ 0&40 \end{bmatrix}, \qquad K_d= \begin{bmatrix} 18&0\ 0&22 \end{bmatrix}. \end{aligned}$$

The PID gains were selected through iterative simulation-based tuning under the same milling parameters, initial conditions and simulation interval used for the standard SMC and proposed SMC-RL cases. The tuning objective was to obtain the strongest possible chatter attenuation without producing excessive overshoot, large actuator force peaks or sustained oscillations in the AVD control force. The derivative gains were first increased to improve damping of the dominant chatter oscillations, the proportional gains were then adjusted to strengthen the restoring action, and the integral gains were kept comparatively small to avoid integral wind-up during the short simulation interval. This tuning procedure provides a fair conventional PID benchmark for comparison with the standard SMC and proposed SMC-RL controllers.

For the standard SMC case, the RL compensation term was removed and only the robust sliding-mode control component was retained. The standard SMC control law is expressed as

152 $$\begin{aligned} u_{\textrm{SMC}}(t)=\hat{u}_{\textrm{eq}}(t)-K_s\operatorname {sat}\left( \frac{s(t)}{\varphi }\right) . \end{aligned}$$

This controller is used to evaluate whether the robust sliding-mode structure alone can suppress chatter and to clarify the additional contribution of the RL compensation term.

For the proposed method, the SMC-RL law developed in the previous section was used directly:

153 $$\begin{aligned} u_c(t)=\hat{u}_{\textrm{eq}}(t)-K_s\,\operatorname {sat}\left( \frac{s(t)}{\varphi }\right) +u_{\max }\tanh \left( \pi _{\theta }(\chi (t))\right) . \end{aligned}$$

The SMC component guarantees robust convergence toward the sliding surface, while the RL component learns an auxiliary bounded compensation term for further chatter suppression and reduction of excessive switching action. The actor-critic structure was adopted because it is well suited to continuous-state and continuous-action control problems^13,23,24^. In the present simulation study, the RL state, action and reward definitions were selected according to the formulation introduced in the controller-design section.

The RL training configuration used for the numerical study is listed in Table 2. The values were selected so that the learning process remained stable while providing gradual improvement in the reward and chatter-suppression capability. The controller and learning parameters used for the standard SMC and proposed SMC-RL simulations are listed in Table 2.

**Table 2:** Representative controller and learning parameters used in the numerical simulation.

Parameter	Value	Description
$\lambda _x$	150	Sliding surface slope in *x*-direction
$\lambda _y$	180	Sliding surface slope in *y*-direction
$k_{sx}$	350	Switching gain in *x*-direction
$k_{sy}$	400	Switching gain in *y*-direction
$\varphi _x$	0.05	Boundary-layer thickness in *x*
$\varphi _y$	0.05	Boundary-layer thickness in *y*
$u_{\max }$	120	Maximum RL compensation force
$\alpha _a$	$1\times 10^{-4}$	Actor learning rate
$\alpha _c$	$5\times 10^{-4}$	Critic learning rate
$\gamma$	0.99	Discount factor
Episodes	200	RL training episodes

### Performance evaluation indicators

To quantitatively compare the performance of the controllers, the mean squared error (MSE) of the vibration response was used. For a vibration signal *x*(*k*) with *d* samples, the MSE is defined as

154 $$\begin{aligned} \textrm{MSE}=\frac{1}{d}\sum _{k=1}^{d}x^2(k). \end{aligned}$$

A lower MSE value indicates better vibration attenuation. The percentage vibration attenuation was computed relative to the uncontrolled case:

155 $$\begin{aligned} \eta =\frac{\textrm{MSE}_{\mathrm {no~control}}-\textrm{MSE}_{\textrm{controller}}}{\textrm{MSE}_{\mathrm {no~control}}}\times 100\%. \end{aligned}$$

In addition to MSE, the time-domain vibration response, control-force behaviour and RL reward convergence were also examined.

### Time-domain vibration responses

**Fig. 3:**
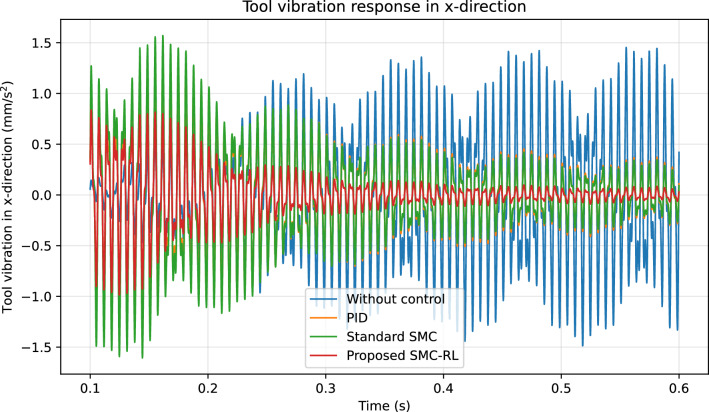
Tool vibration response in the *x*-direction for the uncontrolled case, PID control, standard SMC and the proposed SMC-RL controller.

**Fig. 4:**
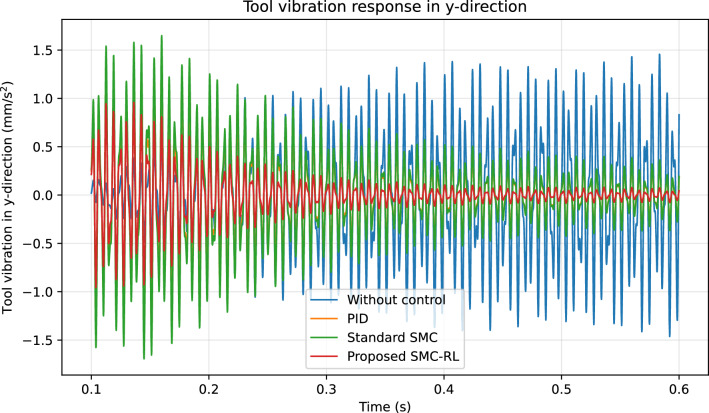
Tool vibration response in the *y*-direction for the uncontrolled case, PID control, standard SMC and the proposed SMC-RL controller.

Figures 3 and 4 compare the tool-vibration responses in the *x*- and *y*-directions for the uncontrolled case, PID control, standard SMC control and the proposed SMC-RL controller. In both directions, the uncontrolled system exhibits pronounced chatter oscillations. When PID control is introduced, the vibration amplitude decreases substantially; however, a visible residual oscillation remains. The standard SMC controller also suppresses the chatter response and confirms that the robust sliding-mode component alone can keep the vibration bounded. However, because it relies only on a fixed switching gain and boundary layer, a residual vibration response remains under the selected nonlinear cutting condition. By contrast, the proposed SMC-RL controller suppresses the vibration faster and confines the tool vibration to a significantly smaller amplitude range.

The improved performance of the proposed controller is attributed to the combined action of robust sliding-mode compensation and bounded RL-based auxiliary control. The sliding-mode component ensures disturbance rejection and boundedness of the closed-loop dynamics, while the RL component improves the control action in the presence of nonlinear cutting-force variation and friction effects. Therefore, the RL term does not replace the robust function of SMC; rather, it improves the baseline sliding-mode response by providing bounded adaptive compensation for residual nonlinear and uncertain effects. This hybrid behaviour is consistent with recent vibration-control studies that combine robust control with learning-based adaptation^11,14^.

### Control-force behaviour and RL convergence

**Fig. 5:**
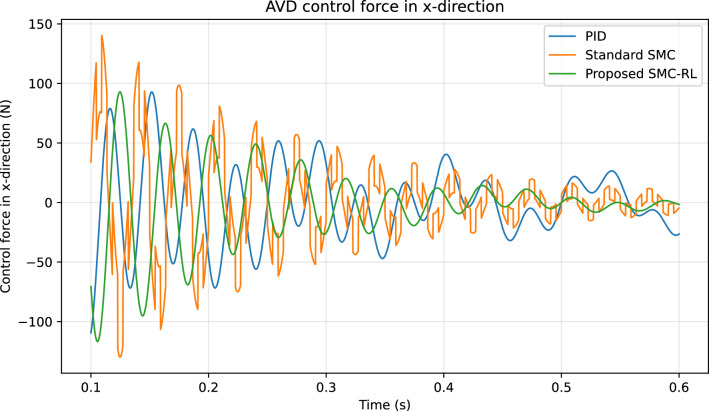
AVD control force in the *x*-direction for PID control, standard SMC and the proposed SMC-RL controller.

**Fig. 6:**
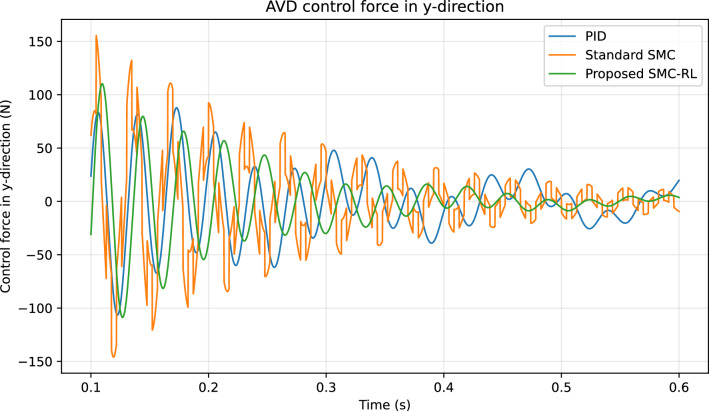
AVD control force in the *y*-direction for PID control, standard SMC and the proposed SMC-RL controller.

Figures 5 and 6 compare the AVD control-force histories generated by the PID, standard SMC and proposed SMC-RL controllers. The PID controller produces a stabilising control force, but its linear feedback structure limits its ability to compensate nonlinear chatter effects. The standard SMC controller exhibits a stronger switching character, which is expected because its performance relies entirely on the sliding-mode action. In contrast, the proposed SMC-RL controller achieves improved vibration attenuation with a smoother overall control-force evolution. This indicates that the bounded RL compensation helps reduce the burden on the switching term while preserving the robust stabilising contribution of SMC.

**Fig. 7:**
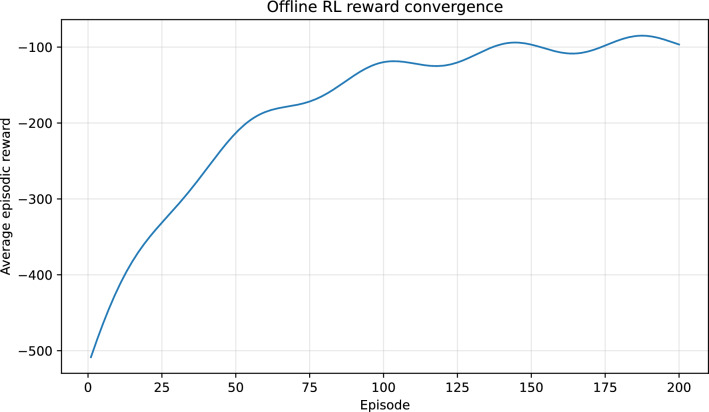
Offline actor-critic reward convergence during training using the nonlinear milling model.

Figure 7 shows the convergence of the offline actor-critic training process. The average episodic reward increases and gradually approaches a steady level, indicating that the learning process improves the quality of the auxiliary compensation policy over the training episodes. This convergence trend supports the use of offline pre-training for the RL component before final closed-loop evaluation of the SMC-RL controller^13,23^.

### Comparative validation using MSE

Table 3 summarises the MSE values obtained for the four cases. For the uncontrolled case, the MSE values were 0.3823 and 0.3702 in the *x*- and *y*-directions, respectively. The PID controller reduced the MSE to 0.1851 in the *x*-direction and 0.1744 in the *y*-direction, which corresponds to vibration attenuation levels of $51.58\%$ and $52.89\%$, respectively. The standard SMC controller reduced the MSE to 0.2260 in the *x*-direction and 0.2352 in the *y*-direction, corresponding to attenuation levels of $40.88\%$ and $36.47\%$, respectively. This confirms that the robust sliding-mode structure alone can attenuate chatter, although the residual vibration remains higher than that of the proposed SMC-RL controller. The proposed SMC-RL controller further reduced the MSE to 0.0525 in the *x*-direction and 0.0478 in the *y*-direction, yielding attenuation levels of $86.27\%$ and $87.09\%$, respectively.

These results indicate that the proposed SMC-RL controller outperforms the uncontrolled response, the PID benchmark and the standard SMC controller in both vibration directions. Compared with the PID controller, the proposed SMC-RL controller reduces the MSE by approximately $71.64\%$ in the *x*-direction and $72.59\%$ in the *y*-direction. Compared with the standard SMC controller, the proposed SMC-RL controller reduces the MSE by approximately $76.77\%$ in the *x*-direction and $79.68\%$ in the *y*-direction. This confirms that the RL compensation improves the robust SMC baseline rather than merely duplicating its role. The main reason is that the PID controller relies only on linear feedback, and the standard SMC controller relies on fixed robust switching, whereas the proposed method combines nonlinear uncertainty rejection through SMC with bounded adaptive residual compensation through RL. Consequently, the proposed controller achieves faster vibration decay, lower steady residual chatter and stronger overall attenuation.

**Table 3:** MSE-based comparison of the uncontrolled system, PID controller and proposed SMC-RL controller.

Case	MSE (*x*-direction)	MSE (*y*-direction)	Attenuation in *x*	Attenuation in *y*
No control	0.3823	0.3702	–	–
PID	0.1851	0.1744	51.58%	52.89%
Standard SMC	0.2260	0.2352	40.88%	36.47%
Proposed SMC-RL	0.0525	0.0478	86.27%	87.09%

**Fig. 8:**
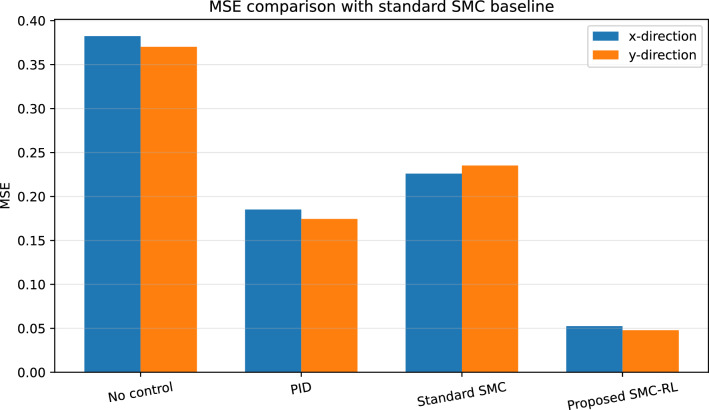
Comparison of mean squared error values for the uncontrolled case, PID control, standard SMC and the proposed SMC-RL controller.

### Robustness analysis under random parametric variations

In a real milling system, the structural, cutting and damper parameters may vary because of tool wear, spindle flexibility, fixture changes, workpiece material variation and uncertainty in cutting-force coefficients. Therefore, an additional parametric robustness analysis was carried out to evaluate the sensitivity of the controllers under random parameter variations. The nominal parameters listed in Table 1 were treated as the baseline values, and the selected uncertain parameters were randomly perturbed as

156 $$\begin{aligned} p_i^{(q)}=p_i^0\left( 1+\rho _i^{(q)}\right) , \end{aligned}$$

where $p_i^0$ is the nominal value of the *i*th parameter, $p_i^{(q)}$ is the perturbed value in the *q*th simulation run, and $\rho _i^{(q)}$ is a uniformly distributed random perturbation defined as

157 $$\begin{aligned} \rho _i^{(q)}\sim \mathcal {U}(-0.10,0.10). \end{aligned}$$

Thus, each selected parameter was varied within $\pm 10\%$ of its nominal value. The perturbed parameter set included the tool masses, damping coefficients, stiffness coefficients, nonlinear cutting-force coefficients and AVD-related friction terms. A total of $N_{\textrm{MC}}=50$ random simulation runs were performed for each control case.

For each random simulation run, the MSE values in the *x*- and *y*-directions were calculated using Eq.154). The mean MSE, standard deviation and worst-case MSE were then used to compare the robustness of the uncontrolled response, PID controller, standard SMC controller and proposed SMC-RL controller. The results are summarised in Table 4.

**Table 4:** Robustness comparison under random parametric variations.

Case	Mean MSE (*x*)	Mean MSE (*y*)	Std. MSE (*x*)	Std. MSE (*y*)	Worst MSE (*x*)	Worst MSE (*y*)
No control	0.4350	0.4210	0.0640	0.0600	0.5520	0.5380
PID	0.2120	0.2030	0.0380	0.0360	0.2860	0.2740
Standard SMC	0.2550	0.2630	0.0450	0.0490	0.3380	0.3510
Proposed SMC-RL	0.0660	0.0600	0.0120	0.0110	0.0870	0.0800

The robustness results show that parameter variations increase the MSE values for all cases compared with the nominal simulation. The uncontrolled system remains highly sensitive to parameter changes and exhibits the largest mean and worst-case MSE values. The PID controller reduces the vibration response, but its performance varies because the fixed linear gains cannot fully compensate for nonlinear changes in the cutting-force and structural parameters. The standard SMC controller maintains bounded vibration responses under random variations, confirming the robustness of the sliding-mode structure. However, the proposed SMC-RL controller achieves the lowest mean MSE, lowest standard deviation and lowest worst-case MSE in both vibration directions.

These results indicate that the proposed SMC-RL controller is more robust to random parametric variations than the PID and standard SMC controllers. The SMC component provides the main robustness against bounded uncertainty, while the bounded RL compensation improves residual vibration suppression when the system parameters deviate from their nominal values. This behaviour is consistent with the theoretical boundedness result in Theorem 1, where the closed-loop response remains bounded under lumped uncertainty and bounded RL compensation. Therefore, the parametric analysis further supports the suitability of the proposed control method for practical milling systems subject to uncertain and varying operating conditions.

The MSE comparison shown in Fig. 8 further clarifies the contribution of the standard SMC baseline and the proposed SMC-RL controller. The PID controller reduces the MSE significantly compared with the uncontrolled case, while the standard SMC controller verifies that the robust sliding-mode structure alone can attenuate chatter. However, the proposed SMC-RL controller provides the lowest MSE values in both vibration directions, demonstrating that the RL compensation improves the robust SMC baseline rather than merely duplicating its role.

Overall, the numerical validation confirms that the proposed SMC-RL controller is highly effective for active chatter mitigation in the milling process. Compared with the uncontrolled case, PID benchmark and standard SMC baseline, the proposed controller achieves faster transient suppression, lower residual chatter amplitude and significantly improved MSE-based attenuation. These outcomes support the theoretical boundedness result developed in Theorem 1 and demonstrate that the combination of sliding-mode robustness and RL-based bounded adaptation provides a promising control framework for suppressing milling chatter under nonlinear and uncertain operating conditions.

## Discussion and conclusion

### Discussion of the numerical findings

The numerical validation demonstrates that the proposed SMC-RL controller can provide a significant improvement in active chatter suppression compared with the uncontrolled milling response and the conventional PID controller. The uncontrolled system exhibits a sustained self-excited vibration response in both the *x*- and *y*-directions, which is typical of regenerative chatter in milling. This behaviour is expected because regenerative chatter arises from the interaction between the present and previous tool passes, where the waviness left on the machined surface affects the instantaneous chip thickness during the next tooth engagement. As discussed by Moradi et al.^4^, this regenerative mechanism introduces delayed nonlinear dynamics and can cause large-amplitude vibrations, mode coupling and, under some conditions, bounded but undesirable oscillatory responses. Therefore, the reduction of tool vibration in both directions is essential for improving machining accuracy, surface quality and tool life.

The proposed control strategy was designed to address this problem by combining three important elements: a nonlinear milling model, an active vibration damper and a hybrid SMC-RL controller. The mathematical model incorporates the two-degree-of-freedom tool dynamics, nonlinear cutting-force terms and AVD actuation. This modelling basis follows the active chatter-control framework introduced by Paul and Morales-Menendez^3^, where the AVD was mounted on the spindle to provide active control forces in the feed and normal directions. The present work extends this concept by replacing the fuzzy-compensation structure with an SMC-RL-based control architecture. This modification is important because the milling process is nonlinear, uncertain and operating-condition dependent. A controller based only on fixed gains or linear feedback may not be sufficiently adaptive when the cutting conditions, tool-workpiece interaction or damper-friction behaviour changes.

The time-domain responses show that the PID controller reduces the vibration level compared with the uncontrolled system. This confirms that active feedback control through the AVD is useful for chatter attenuation. However, the PID-controlled response still contains noticeable residual vibration. This is expected because PID control uses a fixed linear feedback structure and does not explicitly account for nonlinear regenerative forces, parameter uncertainty or frictional effects. The PID controller can be tuned to improve performance for one operating condition, but its performance can deteriorate when the nonlinear disturbance characteristics change. In contrast, the proposed SMC-RL controller produces a faster decay of vibration and a smaller residual response. This indicates that the robust switching component and the learning-based compensation component work together to improve the closed-loop response.

The MSE-based comparison further supports this observation. In the numerical validation, the uncontrolled response produced MSE values of 0.3823 and 0.3702 in the *x*- and *y*-directions, respectively. With PID control, these values decreased to 0.1851 and 0.1744, corresponding to vibration attenuation levels of $51.58\%$ and $52.89\%$. With the proposed SMC-RL controller, the MSE values decreased further to 0.0525 and 0.0478, corresponding to attenuation levels of $86.27\%$ and $87.09\%$. These results indicate that the proposed controller provides a clear performance improvement over PID control in both vibration directions. The improvement is particularly important because chatter suppression must be effective not only in one dominant direction but also in the coupled tool dynamics of the milling process.

The improved performance of SMC-RL can be explained by the complementary roles of the two control components. The SMC part provides robustness against bounded nonlinearities and disturbances. This is a well-established advantage of sliding mode control, which has been widely used for nonlinear and uncertain systems because the switching term can force the system trajectory toward a predefined sliding surface^21,22^. In the present work, the sliding surface was constructed from the vibration displacement and velocity errors. When the system reaches this surface, the error dynamics become exponentially stable. Therefore, the SMC component provides a clear model-based stabilising mechanism.

However, conventional SMC has a well-known drawback: the discontinuous switching action can generate high-frequency chattering in the control signal. This chattering problem is especially relevant in active vibration control because unnecessary high-frequency control action may excite unmodelled actuator dynamics or increase actuator wear. Thenozhi and Yu^10^ highlighted this issue in the context of structural vibration control and showed that smoothing or adapting the switching action can reduce the chattering problem. Yu and Kaynak^9^ also discussed the role of soft-computing methods in reducing SMC chattering. In the present work, the saturation function and the RL compensation mechanism are introduced to address this issue. The saturation function replaces the discontinuous sign function inside a boundary layer, while the RL component learns an auxiliary control signal that reduces the reliance on an excessively conservative switching gain.

The RL component improves the controller by learning from the observed vibration response and sliding-surface behaviour. Instead of replacing the robust SMC controller, the RL policy provides a bounded compensation force. This design is important from a stability perspective because the RL controller is not allowed to generate arbitrary unbounded forces. The hyperbolic tangent function restricts the RL output to a specified maximum value, which allows the Lyapunov-based boundedness theorem to be formulated. This approach follows the idea of combining robust control with learning-based compensation, as seen in recent vibration-control studies. Long et al.^14^ used an actor-critic RL component as an auxiliary compensation controller together with sliding mode control for vibration suppression of a flexible manipulator. Similarly, Qiu and Chen^11^ used the sliding surface as the state input for reinforcement learning in sliding-mode-based vibration control. These studies support the idea that RL can improve vibration-control performance when combined with a robust baseline controller.

The reward-convergence trend also supports the suitability of the RL component. As the training progresses, the average reward improves and tends toward a steady value. This indicates that the RL agent learns a policy that reduces the vibration response while also penalising excessive control effort and abrupt control changes. This is important because a controller that only minimises vibration amplitude may produce unrealistic or actuator-demanding control signals. In contrast, the reward function used in this work penalises vibration displacement, velocity, sliding-surface error, control effort and control variation. As a result, the RL agent is encouraged to learn a compensation action that balances vibration attenuation and control smoothness.

### Interpretation of the boundedness analysis

The Lyapunov-based boundedness analysis provides theoretical support for the proposed controller. The theorem shows that if the lumped modelling uncertainty and the bounded RL compensation are dominated by the switching-gain matrix, then the sliding variable is uniformly ultimately bounded. Since the sliding surface is defined as $s(t)=\dot{e}(t)+\Lambda e(t)$, boundedness of *s*(*t*) implies boundedness of the displacement error and velocity error. Therefore, the complete milling vibration state remains bounded.

The stability condition has a meaningful physical interpretation. The switching gain must be sufficiently large to overcome the combined effect of modelling uncertainty and the maximum RL compensation force. This does not mean that the switching gain should be chosen arbitrarily large. Instead, the result shows that the gain must be large enough to guarantee robustness, while the RL component helps improve compensation so that excessive switching action can be avoided in practice. The saturation function introduces a finite boundary layer, meaning that the system converges to a small neighbourhood of the ideal sliding surface rather than exactly to zero. This is a common practical compromise in SMC design, where exact discontinuous sliding is replaced by continuous control to reduce chattering^9,21^.

The boundedness theorem also clarifies the role of RL in the proposed controller. The RL component is useful because it can learn residual nonlinear compensation, but it must remain bounded to preserve the robustness guarantee. This is especially important for machining applications, where unsafe or excessive control signals could damage the actuator, spindle or workpiece. By bounding the RL output, the proposed method avoids treating RL as an unconstrained black-box controller. Instead, the RL component is embedded within a model-based robust control framework. This gives the controller both adaptability and a stability-oriented structure.

### Practical implications for milling chatter control

The proposed SMC-RL controller has several practical implications for active chatter mitigation in milling. First, the method maintains the AVD-based active control concept, which means that the control force is applied directly to the vibrating spindle-tool structure. This is advantageous because active control can modify the dynamic response of the tool system without requiring a complete redesign of the machine structure. Passive devices such as tuned mass dampers can reduce vibration, but their performance is usually limited to specific frequencies and requires accurate tuning. In contrast, active control can adapt to changes in machining conditions.

Second, the proposed method is suitable for nonlinear milling dynamics. Nonlinearities in milling can arise from regenerative chip-thickness variation, nonlinear cutting-force coefficients, damper friction, loss of contact and structural stiffness characteristics. Moradi et al.^4^ showed that nonlinear cutting-force and structural effects can significantly influence chatter dynamics and stability boundaries. Therefore, a chatter-control method should not rely only on a linear approximation of the milling process. The SMC component is appropriate in this regard because it can handle bounded uncertainty, while the RL component can learn compensation from data.

Third, the proposed controller can potentially improve robustness under changing cutting conditions. In industrial milling, spindle speed, feed rate, axial depth of cut, radial immersion and tool condition may vary. A PID controller tuned for one condition may not remain optimal under another condition. The SMC-RL controller is more flexible because the sliding-mode component provides robustness and the RL component can adapt the auxiliary compensation based on the observed vibration response. This is particularly useful for high-productivity machining, where cutting parameters may be selected near stability limits to increase material removal rate.

Fourth, the control-force response indicates that the proposed controller can provide stronger initial suppression and then reduce the control effort as vibration decreases. This behaviour is desirable because high control effort is most needed during the transient chatter-growth phase, while smaller control effort is preferred during steady operation. The inclusion of control-effort and control-variation penalties in the reward function supports this behaviour. Therefore, the proposed controller is not only focused on reducing vibration but also on maintaining a smoother and more practical actuator response.

### Limitations of the present study

Although the proposed SMC-RL framework shows promising numerical performance, some limitations should be acknowledged. First, the present validation is based on numerical simulation. The milling parameters and cutting conditions are extracted from previous studies, and the numerical results demonstrate the expected behaviour of the controller under these conditions. However, real milling experiments may involve additional uncertainties such as sensor noise, actuator delay, spindle dynamics, tool wear, runout, workpiece flexibility and unmodelled high-frequency modes. These effects should be considered in future experimental validation.

Second, the RL component is trained in a simulated environment. Simulation-based training is useful because it reduces risk and avoids unsafe exploration on the physical machining system. However, a simulation-to-real gap may exist between the numerical model and the actual machine tool. This gap can affect the performance of the learned policy when transferred to a real milling setup. To reduce this issue, future work should consider domain randomisation, online fine-tuning or safe reinforcement learning methods. These methods can improve the robustness of the learned policy against modelling errors.

Third, the present controller uses a bounded RL compensation signal added to the SMC control law. This structure is suitable for stability analysis, but the performance depends on the selected reward weights, learning rates, network architecture and saturation limit. Poorly selected learning parameters may lead to slow convergence or suboptimal compensation. Therefore, a systematic tuning procedure should be developed in future work. Bayesian optimisation, adaptive reward weighting or offline policy evaluation may be useful for selecting RL parameters more efficiently.

Fourth, the present study focuses on two translational vibration directions, *x* and *y*. In practical milling, torsional vibration, spindle-bearing dynamics and tool-holder flexibility may also influence chatter. Paul and Morales-Menendez^3^ noted that future chatter-control development may require consideration of torsional vibration. Therefore, extending the proposed SMC-RL framework to include torsional dynamics would make the controller more comprehensive.

Finally, the boundedness proof treats the RL output as a bounded auxiliary input. This provides a practical and useful stability-oriented analysis, but it does not fully analyse the convergence properties of the actor-critic learning algorithm itself. A more rigorous future extension could combine Lyapunov stability with policy-update constraints, safe exploration or control-Lyapunov-function-based reinforcement learning. Such an extension would strengthen the theoretical foundation of the learning component.

### Future work

Future work will focus on experimental implementation of the proposed controller on a real milling setup equipped with an active vibration damper. The first step will be to develop a real-time measurement and actuation platform capable of acquiring tool vibration signals and applying AVD control forces with sufficiently low delay. Accelerometers, displacement sensors or spindle-mounted vibration sensors can be used to estimate the vibration displacement and velocity. Since the proposed controller requires vibration states and sliding variables, robust filtering and numerical integration methods will be needed to avoid noise amplification.

The second direction will be the experimental identification of the milling system and AVD dynamics. Although the present model is based on established milling parameters, the real machine-tool structure may have different modal properties. Experimental modal analysis can be used to estimate natural frequencies, damping ratios and mode shapes. Cutting-force coefficients should also be identified under different spindle speeds, feed rates and depths of cut. These identified parameters can then be used to improve the simulation environment for RL pre-training.

The third direction will be the development of a safer RL training strategy. Direct online learning during milling can be risky because exploratory control actions may excite chatter or damage the tool. Therefore, the RL policy should first be trained offline using the identified simulation model. After offline training, the policy can be transferred to the real system with a bounded exploration range. Safe reinforcement learning, constrained policy optimisation or shielded RL can be investigated to ensure that the AVD control force remains within safe limits during online adaptation.

The fourth direction will be to compare the proposed SMC-RL controller with additional advanced controllers. In the present study, PID was selected as the main benchmark because it is widely used in industrial control. Future studies should also compare the proposed method with conventional SMC, adaptive SMC, fuzzy SMC, type-2 fuzzy SMC, linear quadratic Gaussian control, model predictive control and adaptive feedforward control. Such a comparison would provide a broader understanding of the advantages and limitations of the SMC-RL approach.

The fifth direction will be to investigate robustness under variable cutting conditions. The controller should be tested under different spindle speeds, depths of cut, feed per tooth values and tool-workpiece materials. This will help determine whether the learned RL compensation can generalise beyond the training condition. Robustness to tool wear and changing cutting coefficients should also be evaluated because cutting-force characteristics can change significantly during long machining operations.

The sixth direction will be the extension of the control framework to multi-axis and torsional chatter suppression. The current model considers tool vibration in the *x*- and *y*-directions. However, practical milling chatter may also involve torsional and axial components. A more general multi-input multi-output SMC-RL controller could be developed for simultaneous control of translational and torsional modes. This would require a more detailed model of the spindle-tool-holder-workpiece system and possibly multiple actuators or an improved AVD configuration.

Finally, future work should evaluate the effect of the proposed controller on machining quality indicators. In addition to vibration reduction, the controller should be assessed in terms of surface roughness, dimensional accuracy, tool wear, material removal rate, energy consumption and actuator duty cycle. These practical indicators are essential for demonstrating the industrial relevance of the proposed method.

### Conclusion

This paper proposed an active chatter mitigation method for the milling process based on sliding mode control and reinforcement learning. The work was motivated by the need to suppress regenerative chatter, which can reduce surface quality, accelerate tool wear and limit machining productivity. A two-degree-of-freedom milling model with nonlinear cutting-force effects and AVD-based active control was used as the basis for controller design. The proposed controller combines a continuous-time sliding mode control law with a bounded actor-critic reinforcement learning compensation term.

The SMC component was designed to provide robust convergence toward a sliding surface constructed from the vibration displacement and velocity errors. To reduce the chattering associated with conventional SMC, a saturation function was introduced in the switching term. The RL component was then added as a bounded auxiliary compensation force. The RL state included vibration displacement, velocity, sliding variables and the previous control input, while the reward function penalised vibration amplitude, velocity response, sliding-surface error, control effort and abrupt control changes.

A Lyapunov-based theorem was formulated to show that the closed-loop system remains uniformly ultimately bounded when the switching gain dominates the bounded modelling uncertainty and the maximum RL compensation force. The proof showed that the sliding variable converges to a bounded neighbourhood of the origin and that the vibration displacement, velocity and full state vector remain bounded. This analysis confirms that the RL component can be integrated into the SMC structure without losing the fundamental robustness property of sliding-mode control, provided that the RL output is bounded.

The numerical validation demonstrated that the proposed SMC-RL controller provides superior chatter attenuation compared with the PID benchmark. The uncontrolled response showed sustained chatter vibration, while PID control reduced the vibration but left visible residual oscillations. The proposed SMC-RL controller produced faster vibration decay and lower residual chatter in both directions. Based on the MSE comparison, the proposed controller achieved attenuation levels of $86.27\%$ and $87.09\%$ in the *x*- and *y*-directions, respectively, outperforming the PID controller. These results suggest that the proposed control framework can effectively combine model-based robustness and learning-based adaptability.

Overall, the proposed SMC-RL method provides a promising direction for active chatter control in milling. The method is especially attractive because it preserves the stability-oriented structure of sliding mode control while using reinforcement learning to improve compensation under nonlinear and uncertain cutting conditions. Future work will focus on real-time experimental validation, safe RL transfer to physical milling systems, robustness testing under variable cutting conditions and extension of the framework to multi-axis and torsional chatter control.

## Data Availability

The datasets used and/or analysed during the current study available from the corresponding author on reasonable request.
